# Reduced GLP-1R availability in the caudate nucleus with Alzheimer’s disease

**DOI:** 10.3389/fnagi.2024.1350239

**Published:** 2024-06-10

**Authors:** Emma Barrett, Gabrielle Ivey, Adam Cunningham, Gary Coffman, Tyera Pemberton, Chan Lee, Prabir Patra, James B. Day, Peter H. U. Lee, Joon W. Shim

**Affiliations:** ^1^Department of Biomedical Engineering, Marshall University, Huntington, WV, United States; ^2^Department of Anesthesia, Indiana University Health Arnett Hospital, Lafayette, IN, United States; ^3^Department of Orthopedic Surgery, Cabell Huntington Hospital and Marshall University School of Medicine, Huntington, WV, United States; ^4^Department of Cardiothoracic Surgery, Southcoast Health, Fall River, MA, United States; ^5^Department of Pathology and Laboratory Medicine, Brown University, Providence, RI, United States

**Keywords:** chronic hydrocephalus, Alzheimer’s disease, GLP-1R, DCX, hemoglobin

## Abstract

The glucagon-like peptide-1 receptor (GLP-1R) agonists reduce glycated hemoglobin in patients with type 2 diabetes. Mounting evidence indicates that the potential of GLP-1R agonists, mimicking a 30 amino acid ligand, GLP-1, extends to the treatment of neurodegenerative conditions, with a particular focus on Alzheimer’s disease (AD). However, the mechanism that underlies regulation of GLP-1R availability in the brain with AD remains poorly understood. Here, using whole transcriptome RNA-Seq of the human postmortem caudate nucleus with AD and chronic hydrocephalus (CH) in the elderly, we found that GLP-1R and select mRNAs expressed in glucose dysmetabolism and dyslipidemia were significantly altered. Furthermore, we detected human RNA indicating a deficiency in doublecortin (DCX) levels and the presence of ferroptosis in the caudate nucleus impacted by AD. Using the genome data viewer, we assessed mutability of GLP-1R and 39 other genes by two factors associated with high mutation rates in chromosomes of four species. Surprisingly, we identified that nucleotide sizes of GLP-1R transcript exceptionally differed in all four species of humans, chimpanzees, rats, and mice by up to 6-fold. Taken together, the protein network database analysis suggests that reduced GLP-1R in the aged human brain is associated with glucose dysmetabolism, ferroptosis, and reduced DCX+ neurons, that may contribute to AD.

## Introduction

Alzheimer’s disease (AD) is the seventh leading cause of mortality globally and #1 cause of dementia (Knopman et al., 2021; Scheltens et al., 2021; Nandi et al., 2024). Age, family history, and genetics followed by high blood *sugar* or diabetes are the largest risk factors for AD (Profenno et al., 2010; Butterfield and Halliwell, 2019; Yiannopoulou and Papageorgiou, 2020). The estimated total cost of AD for 2022 is $321 billion, an expense projected to increase to more than $1 trillion by 2050 (Skaria, 2022). AD is molecularly characterized by plaques of amyloid beta (aβ) and neurofibrillary tangles of tau (Blennow et al., 2006). Mutations in the amyloid precursor protein (APP) and presenilin genes (Boutajangout et al., 2004), both linked to aβ metabolism cause familial AD, a very rare autosomal dominant disease with early onset (Catania et al., 2022; Kalfon et al., 2022; Pagnon de la Vega et al., 2022; Hebestreit et al., 2023; Kriebs, 2023; Lardelli et al., 2023; Li et al., 2023). In most cases, however, sporadic AD is more common with roughly 15 million people affected worldwide (Almkvist and Nordberg, 2023; Ansari et al., 2023; Sepulveda-Falla et al., 2023). The risk of developing AD is influenced by heritable factors to the extent of 60–80% (Mez et al., 2016; Bao et al., 2022; Karlsson et al., 2022), and more than 40 genetic risk loci associated with AD have been identified (Kunkle et al., 2019; Sullivan et al., 2022; Dato et al., 2023; Fominykh et al., 2023; Huang et al., 2023; Wainberg et al., 2023). Among these loci, apolipoprotein E (APOE) alleles exhibit the strongest association with the disease (Park et al., 2023; Polsinelli et al., 2023; Sadleir and Vassar, 2023). Advanced biomarkers, such as positron emission tomography (PET) scans and plasma assays for aβ and phosphorylated tau, demonstrate significant potential for both clinical and research applications (Feinkohl et al., 2020; Peng et al., 2021; Shen et al., 2022; Wilson et al., 2022; Gonzalez-Ortiz et al., 2023; Zhang et al., 2023).

Glucagon-like peptide-1 receptor (GLP-1R) is a G-protein coupled receptor for glucagon-like peptide-1 (GLP-1) (Sloop et al., 2018), a 30 amino acid peptide or hormone released by the intestines in response to food intake (Singh et al., 2022). GLP-1R became a drug target as part of the incretin concept in a search for insulin-stimulating factors for more than 100 years (Holst, 2019). The natural form of GLP-1 undergoes degradation within approximately 2–3 min in the bloodstream. Consequently, various GLP-1 receptor agonists have been developed to extend their *in vivo* effects. These agonists pertain to short-acting compounds, including exenatide (exendin-4), a 39 amino acid peptide whose sequence is 53% homologous to GLP-1, originally isolated from the Gila monster (Graham et al., 2020), which result in brief receptor activation, and long-acting compounds that ensure continuous GLP-1R activation (Meier, 2012). For GLP-1R to continuously activate, either GLP-1 or GLP-1R agonist that mimics the actions of GLP-1 is expected to have sufficient availability in GLP-1R expressing cells. The mRNA for GLP-1 receptors has been identified in various bodily regions and it is the nucleus tractus solitarius (NTS) in the brainstem, which can synthesize GLP-1 in addition to gut (Yildirim Simsir et al., 2018). Dyslipidemia, marked by irregular lipid levels including low-density lipoprotein (LDL) (Bosso et al., 2022; Higashi, 2023) and low-density lipoprotein receptor adapter protein 1 (LDLRAP1) (Ahangari et al., 2021) in the bloodstream, has been proposed as potentially linked to a heightened risk of AD (Oliveira et al., 2018; Wang et al., 2022). The connection between dyslipidemia and Alzheimer’s is not completely comprehended and that GLP-1 has been shown to protect against dyslipidemia (Patel et al., 2014, 2017, 2018; Jall et al., 2017) and promote neurogenesis (McGovern et al., 2012; Lennox et al., 2013; Bae and Song, 2017).

The caudate nucleus is a key component of the basal ganglia that regulates motor control, learning and memory, reward and motivation, and executive functions. The imaging study shows that amyloid imaging marker AV-45 is elevated in the caudate nucleus and putamen of late-onset AD (Kim et al., 2022). Recent research suggests a connection between the caudate nucleus and AD through atrophy and reduced volume, potential role in early detection, disrupted function and symptoms, difficulty with movement coordination, problems with learning and memory, and apathy and emotional dysregulation (Almeida et al., 2003; Madsen et al., 2010; Udo et al., 2020). Neurogenesis, the process of generating new neurons, was traditionally believed to be limited to the embryonic and early postnatal stages in the development of the central nervous system. However, more recent research has challenged this view, suggesting that certain brain regions, including the caudate nucleus, may exhibit neurogenesis to some extent in adulthood (Ernst et al., 2014; Ernst and Frisen, 2015). Doublecortin (DCX) (Salvi et al., 2016), expressed in immature neurons, is one of the markers for postnatal neurogenesis (Mirzadeh et al., 2010; Shahsavani et al., 2018).

The aim of this study was to test the hypothesis that aging with sustained glucose intake, which reduces availability of cerebral GLP-1R, contributes to cognitive decline of the brain, leading to CH and/or AD depending on functionality of metabolic clearance. In doing so, mutability of GLP-1R and neighboring genes might differ depending on species as measured by two factors associated with high mutation rates in human chromosomes: (i) proximity to telomeres, and (ii) high adenine and thymine (A + T) content, since full blown spectrum of cognitive decline as seen in humans with AD is reported to be different in the said primate or Chimpanzees (Sherwood et al., 2011; Lacreuse et al., 2020). Given dopamine receptor D2 (DRD2) being well-conserved during evolution unlike serotonin receptor, 5-hydroxytryptamine receptor 2A (HTR2A), the full-length sizes of the incretin receptor in four species are assessed as well to determine how evolutionarily conserved or advanced receptor GLP-1R might be in mouse, rat, chimpanzee, and human chromosome.

## Results

**Figure 1:**
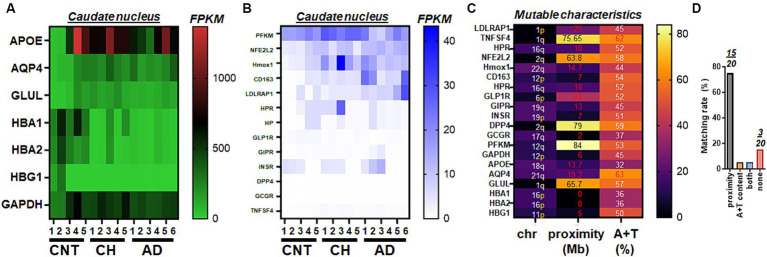
Select gene expressions from whole transcriptome RNA-Seq of human postmortem brains. **(A)** The heat map illustrating an overall view of whole transcriptome RNA-Seq, represented by genes mediating glucose metabolism (GAPDH, GLUL), genetic predispositions to AD (APOE), glymphatic function (AQP4), and hemoglobin (HBA1, HBA2, HBG1) status in control (CNT, *n* = 5), chronic hydrocephalus (CH, *n* = 5), and Alzheimer’s disease (AD, *n* = 6). **(B)** The heat map illustrating an overall view of whole transcriptome RNA-Seq, represented by genes mediating glucose (PFKM, GLP-1R, GIPR, INSR, DPP4 and GCGR), inflammation (TNFSF4), ferroptosis (HPR, NFE2L2, Hmox1, CD163, and HP), and cholesterol (LDLRAP1) metabolism in CNT (*n* = 5), CH (*n* = 5), and AD (*n* = 6). For clarity, plots are grouped by the order of magnitude in RNA amounts (**A**, high RNA; **B**, low RNA). **(C)** Mutable characteristics quantified by two factors of proximity to telomeres and high A + T content associated with high mutation rates in human chromosomes. Mb, million bases. **(D)** Matching rates of either of the two factors and 20 genes shown in **(A–C)**.

Using whole transcriptome RNA-Seq (refer to the method for details), the top 20 were categorized based on the size of nucleic acid fragments. One group (comprising 7 genes) exhibited relatively higher RNA fragments, with FPKM >100 (“high RNA”), including APOE and hemoglobin subunit alpha 1 (HBA1). The other group (comprising 13 genes) showed relatively lower RNA levels, with FPKM <100 (“low RNA”), such as phosphofructokinase, muscle (PFKM) and GLP-1R. The high RNA group, which includes APOE, indicated that genes encoding hemoglobin subunit proteins like HBA1 and hemoglobin subunit alpha 2 (HBA2), exhibited consistent transcript levels across different diagnoses, including control, CH in the elderly, and/or AD (Figure 1A). Despite the clear detectability of mRNA levels, aquaporin 4 (AQP4) and glutamate-ammonia ligase (GLUL) did not exhibit differences in CH or AD when compared to control specimens. The compilation of genes featuring lower FPKM, which includes PFKM, implies that LDLRAP1 might elevate specifically in AD (Figure 1B). Given the 20 candidate genes of interest, we assessed their genomic characteristics of the two factors associated with high mutation rates over human chromosomes, i.e., (i) proximity to telomeres, and (ii) high A + T content (Figure 1C). We found that 15 of 20 human genes screened during whole transcriptome RNA-Seq satisfied proximity to telomeres while three genes (NFE2L2, PFKM, and GLUL) failed to meet either of the two factors (Figure 1D). The two factor analyses on 10 clonal hematopoiesis-driver genes and 10 loci associated with copy number variations suggested that protein tyrosine phosphatase non-receptor type 11 (PTPN11) (Supplementary Figure S1) might be associated with hemoglobin change (Figures 1A,B; Supplementary Tables S1–S3).

**Figure 2:**
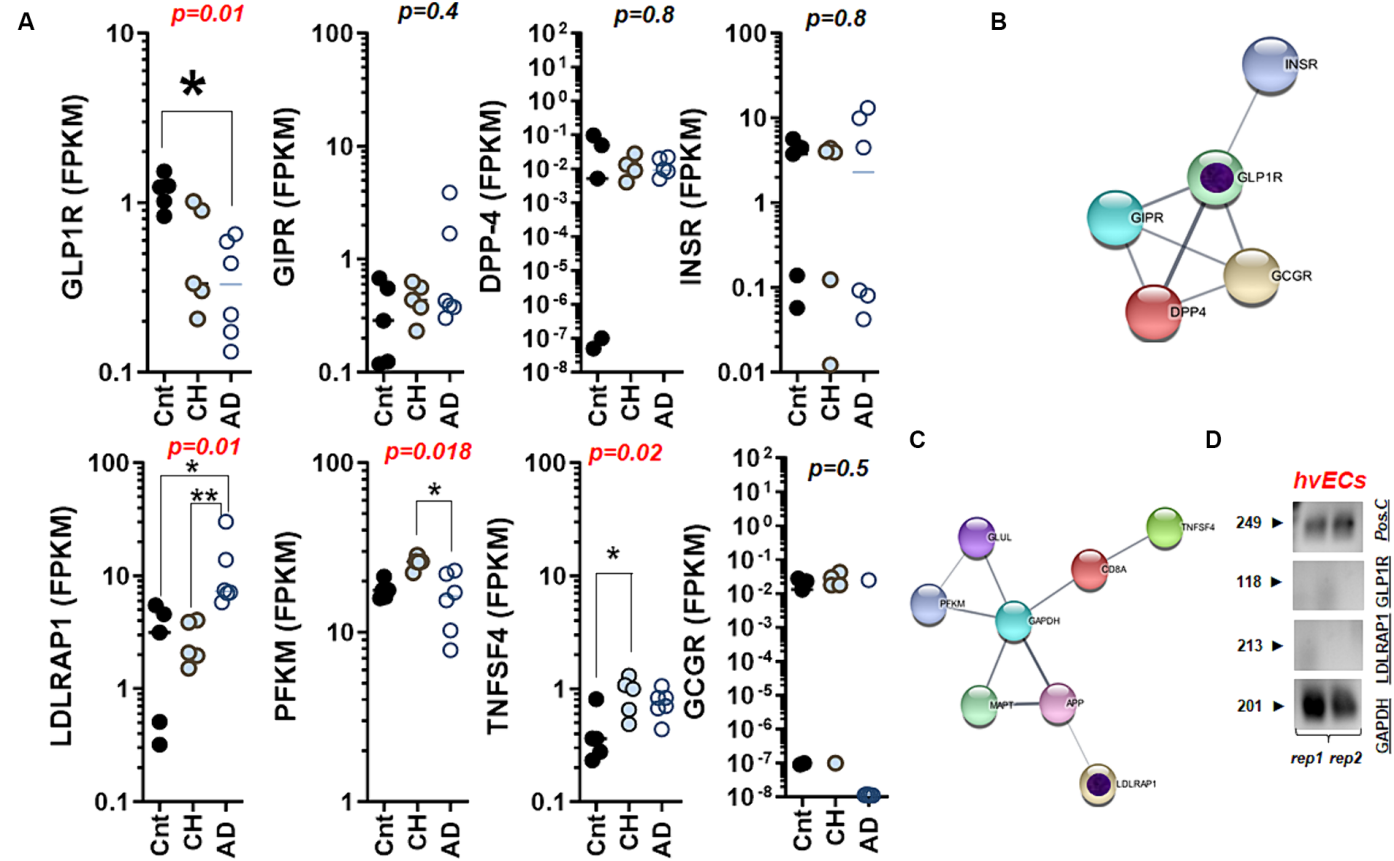
Differential regulations of GLP-1R and LDLRAP1 in the aged brain with CH and AD. **(A)** The scatter plots summarizing glucose and cholesterol dysmetabolism through mRNA levels of GLP-1R, GIPR, DPP4, INSR, LDLRAP1, PFKM, TNFSF4, and GCGR in the caudate nucleus with CH and AD as compared to those of control (Cnt) obtained from the whole transcriptome RNA-Seq; CH, chronic hydrocephalus; AD, Alzheimer’s disease; statistical analysis by Kruskall Wallis test. **(B)** A network chart showing associations between GLP-1R and genes mediating glucose-driven insulin secretion depicted in **(A)**. Note that these GLP-1R-related genes show detectable levels of FPKM in the brain (the caudate nucleus) except DPP4 (almost zero). **(C)** A network chart showing associations between LDLRAP1 and genes mediating inflammation, glucose, and cholesterol metabolism depicted in **(A)**. Putative core genes for AD marked with inner circles in purple **(B,C)**. **(D)** Agarose gels displaying absence of GLP-1R and LDLRAP1 transcripts in human vascular endothelial cells (hvECs) line. Pos.C, positive control with the known molecular size at 249 bp. rep., replicate. *p < 0.05; **p < 0.01 by Dunn’s multiple comparisons after Kruskal-Wallis ANOVA.

Next, we assessed genes encoding the incretin and related molecules, which regulate glucose-dependent insulin secretion. We found that GLP-1R, glucose-dependent insulinotropic polypeptide receptor (GIPR), and insulin receptor (INSR) gene were detected at 1–10 FPKM and that GLP-1R was significantly decreased in the caudate nucleus with AD as compared to that of unaffected controls (*p* = 0.01). However, the transcript of dipeptidyl peptidase 4 (DPP4) and glucagon receptor (GCGR) were neither significantly different nor higher than 1 FPKM, suggesting that expression levels of these two genes, reported to be expressed in gut and/or liver, were low in the aged brain (Figures 2A,B). Consistent with the heatmap of RNA-Seq (Figure 1), we found that LDLRAP1 was significantly elevated in the caudate nucleus with AD (*p* = 0.01) as compared to that of age-matched controls, while tumor necrosis factor (ligand) superfamily, member 4 (TNFSF4) was only elevated in the caudate nucleus with CH (*p* = 0.02). Among seven human genes forming a protein network with LDLRAP1, PFKM gene demonstrated a significant difference (*p* = 0.018) between CH and AD (Figures 2A,C). To test an idea if GLP-1R or LDLRAP1 is expressed in vascular endothelial cells, we assayed these genes along with the positive control gene and GAPDH as internal reference. Consistent with the prior report (Lovshin and Cherney, 2015), gel electrophoresis following RT-PCR indicated that the mRNA for GLP-1R and LDLRAP1 were not detectably expressed in the human vascular endothelial cell line (Figure 2D; Supplementary Figure S2).

**Figure 3:**
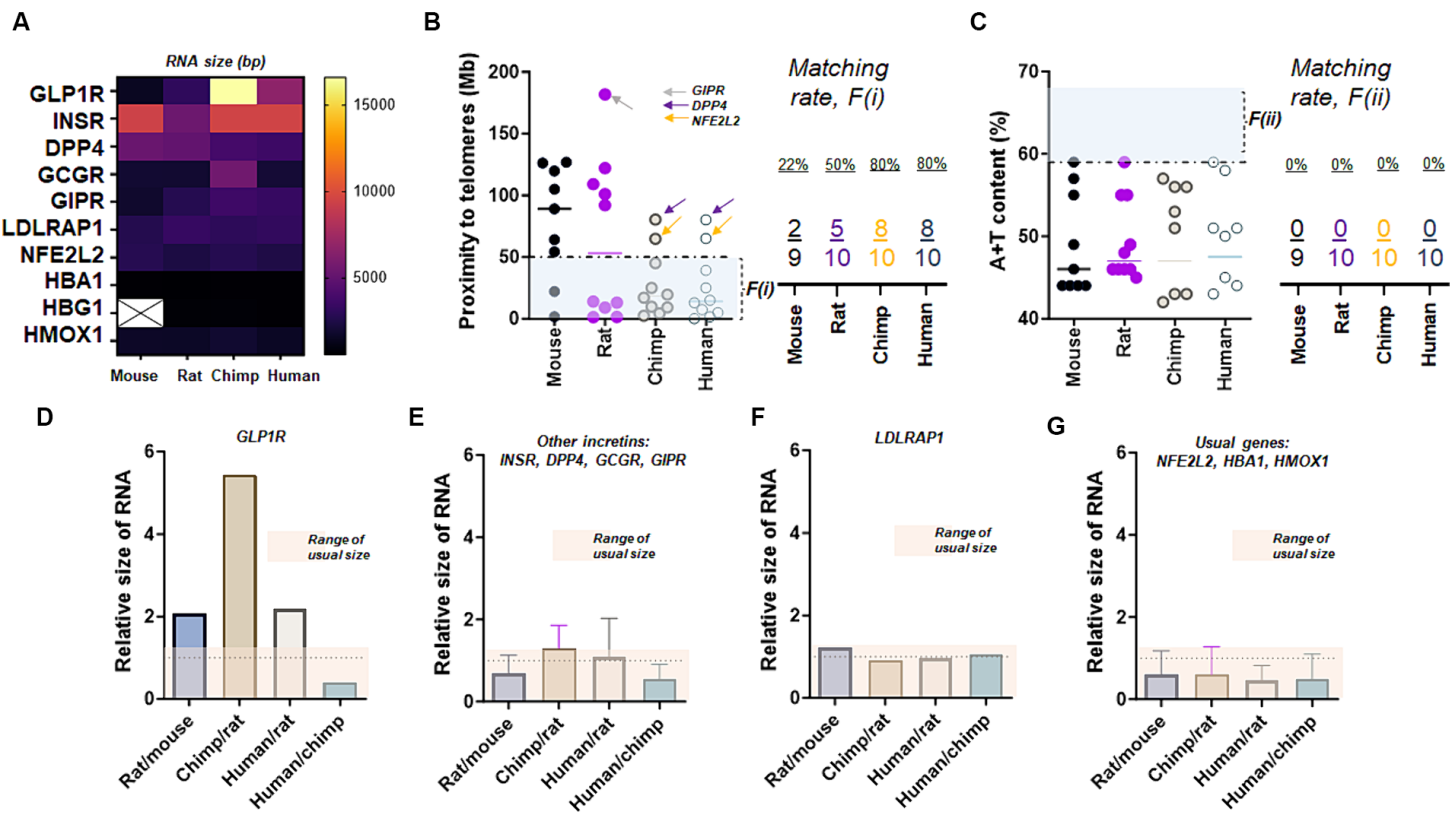
Exceptional RNA sizes of GLP-1R. **(A)** The transcript (RNA) size of GLP-1R and nine other genes over four species. Note that the nucleotide sizes of GLP-1R differ in all four species while there is no HBG1 transcript detected in mouse chromosomes at the genome data viewer. **(B)** Proximity to telomeres of 10 genes over four species. Note that GLP-1R except two other genes (DPP4 and NFE2L2) shown here have evolved in a way meeting proximity to telomeres or the first factor, F(i), associated with high mutation rate as eight human genes are at less than 50 Mb as compared to those of mice and rats. **(C)** A + T content of 10 genes over four species. Ten genes shown here demonstrate a similar characteristic of difficulty in meeting this second factor, F(ii), associated with high mutation rate. **(D)** Bar graph summarizing relative sizes of the transcript (RNA), suggesting unusual variations in GLP-1R over fours species. **(E)** Bar graph showing relative sizes of the transcript (RNA) in four incretin genes other than GLP-1R, suggesting unusual diversion over rat, chimpanzee, and human chromosome. **(F)** Bar graph showing relatives sizes of LDLRAP1 transcript (RNA) over four species. **(G)** Bar graphs showing relatives sizes of typical transcripts over four species.

Examining two factors linked to high mutation rates in these genes, we observed that GLP-1R and the genes related to insulin exhibit exceptional RNA sizes. We conducted a comparison of the transcript sizes of molecules associated with the incretin, including GLP-1R and five others like LDLRAP1, across four different species. The results indicate that GLP-1R exhibits an unusually longer transcript length in chimpanzees, and there is no consistent nucleotide length observed across mouse, rat, chimpanzee, and human (Figure 3A). This finding is moderately akin to a serotonin receptor, where there is approximately a 3.6-fold difference in size between rat (1,566 bp) and chimpanzee HTR2A (5,787 bp). GLP-1R and incretins except GIPR satisfied proximity to telomeres at <50 Mb, while DPP4 and NFE2L2 failed to meet proximity to their telomeres or F (i) (Figure 3B). However, all 10 genes did not satisfy high A + T content at >59% (Figure 3C). The unusual variations of GLP-1R transcripts over four different species were evident as we compared the relative sizes of RNA via comparisons of Rat/mouse, Chimp/rat, Human/rat, and Human/chimp (Figure 3D). Such an exceptional molecular size was also found in incretins and associated genes to a lesser extent as we compared INSR, DPP4, GCGR, and GIPR collectively over four species (Figure 3E). In contrast to GLP-1R and incretins, LDLRAP1 exhibited uniform RNA sizes across the mouse, rat, chimpanzee, and human genomes (Figure 3F). This is in contrast to other genes outside the incretin family, such as NFE2L2, HBA1, and HMOX1 (Figure 3G). This pattern is reminiscent of a dopamine receptor, where the nucleotide size of the mouse (2,778 bp) and human DRD2 (2,808 bp) is nearly identical. Moreover, the caudate nucleus in elderly individuals with AD displayed a deficiency in doublecortin (DCX) (Supplementary Figure S3A) and indicated the loss of the marker for axonal injury or tubulin beta class I (TUBB) (Supplementary Figure S3). This observation is further substantiated by a declining trend in the gene expression of tubulin beta 1 class VI (TUBBP1) in the caudate nucleus (Supplementary Figure S4).

**Figure 4:**
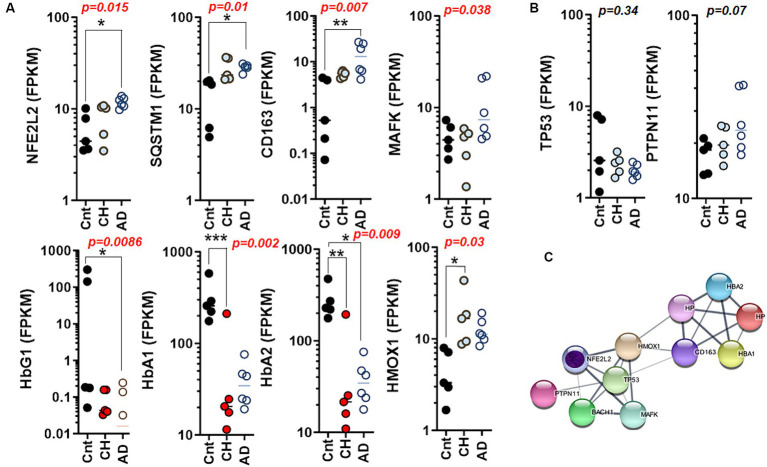
Differential regulation of autophagy and ferroptosis marker genes in the aged brain with CH and AD. **(A)** The scatter plots summarizing autophagy, ferroptosis, and iron homeostasis through mRNA levels of NFE2L2, SQSTM1, CD163, MAFK, HBG1, HBA1, HBA2, and HMOX1 in the caudate nucleus with CH and AD as compared to those of control (Cnt) obtained from the whole transcriptome RNA-Seq. **(B)** The scatter plots summarizing hematopoiesis-driver genes through mRNA levels of TP53 and PTPN11 in the caudate nucleus with CH and AD as compared to those of Cnt obtained from the whole transcriptome RNA-Seq; statistical analysis by Kruskal–Wallis test **(A,B)**. **(C)** A network chart showing interconnections and association between autophagy (NFE2L2) and genes mediating ferroptosis depicted in **(A)**. Note that TP53 and PTPN11 are the mediators linking NFE2L2 (autophagy/ferroptosis) to hemoglobin/iron homeostasis as hematopoiesis driver genes. A putative core gene for AD marked with inner circle in purple **(C)**. *p < 0.05; **p < 0.01; ***p < 0.005 by Dunn’s multiple comparisons after Kruskal-Wallis ANOVA.

Next, we assessed the levels of genes encoding hemoglobin subunit proteins as GLP-1R is associated with glycated hemoglobin (HBA1c). Strikingly, the caudate nucleus affected by AD exhibited elevated levels of the transcription factor NFE2L2 (a marker for oxidative stress/autophagy/ferroptosis), SQSTM1 (indicative of autophagy and inflammation), and CD163 (a marker for macrophage presence or microglial activation) (Figure 4A). The Kruskal–Wallis one-way analysis of variance test revealed a significant difference in the median of MAF BZIP transcription factor K (MAFK), which is a marker for oxidative stress and inflammation, among the control, CH, and AD groups. On the other hand, hemoglobin subunit gamma 1 (HBG1) and HBA2 gene were significantly decreased in the caudate nucleus with AD. HBA1 and HBA2 exhibited a significant reduction in the caudate nucleus of elderly individuals with CH. We also examined the condition of genes associated with clonal hematopoiesis, but we did not observe any significant differences in the expression of these genes in the caudate nucleus with CH and/or AD (Figure 4B). Collectively, NFE2L2 is intricately connected with HMOX1, TP53, MAFK, BTB and CNC homology 1 (BACH1), and PTPN11. In contrast, the network involving HBA1, haptoglobin (HP), CD163, and HBA2 is linked through HMOX1 (Figure 4C).

Collectively, we found that genes encoding four separate protein networks involving heme or hemoglobin cluster (Figure 1A), GLP-1R or incretin cluster (Figure 1B), inflammatory response or LDLRAP1 cluster (Figures 1B,C, 2, 3), and autophagy cluster (Figure 4) can be linked when GAPDH is added as a linker molecule connecting each network (Supplementary Figure S5). To validate unbiased study results on RNA-Seq, PCA analyses providing variances of the dataset (Supplementary Figures S6, S7), GSEA on AD (Supplementary Figure S8) and CH (Supplementary Figure S9) along with enrichment analyses via G-profiler (Supplementary Figures S11, S12), Hierarchical Clustering (Supplementary Figure S13), and histological detection of vascular proteins as compared to RNA-Seq dataset are conducted as provided in Supplementary Figure S14.

## Discussion

Late-onset AD, which usually develops in individuals age mid-60s, affects 90–95% of all Alzheimer’s diagnoses and arises from brain alterations that develop over a long period due to aging (Reitz et al., 2020). In this study, we provided comprehensive RNA-Seq data for elderly postmortem specimens (with a median age of approximately 75 years), supporting the hypothesis that AD is marked by reduced levels of both GLP-1R and DCX. This implies a potential association with glucose dysmetabolism and compromised neurogenesis in the caudate nucleus. In addition to the role of DCX in neurogenesis, it has been demonstrated that neural stem cells, with absent or reduced DCX protein expression, exhibit impaired migration, delayed differentiation and deficient neurite formation (Shahsavani et al., 2018). To enhance cognitive function using pharmaceutical intervention, these findings strongly imply that solely inhibiting cerebral amyloid plaques may fall short. Achieving the restoration of robust connections, which involves neuronal projections from cell bodies in the NTS and/or hindbrain to the basal ganglia, may necessitate the reinstatement of GLP-1R-expressing neurons or the correction of deficient levels of DCX. This connectivity is crucial for cognition involving learning and memory processes in conjunction with the hippocampus.

Despite updates to the classical Hardy-Allsop “amyloid hypothesis” (Hardy and Allsop, 1991), the mechanistic link(s) between *sugar* (*glucose*) intake and cognitive function remain to be fully elucidated. Typically, in subjects with normal blood *sugar* or non-diabetes systemic blood glucose homeostasis in humans is under the control of glucagon-like peptide-1(7–36)amide (GLP-1), a peptide secreted from intestinal enteroendocrine L cells in response to a meal. Previously, in mice lacking GLP-1 receptor (GLP-1R), interactions between diabetes and AD have been suggested, revealing the phenotype with impaired synaptic plasticity and memory formation (Abbas et al., 2009). Our RNA-Seq data presented in this study suggests that GLP-1R mRNA levels were decreased in the caudate nucleus with AD. Taken together, reduced GLP-1R in the basal ganglia of the aged brain is associated with cognitive decline in AD through dysfunctional clearance of amyloid.

Given that monoclonal antibodies approved by the United States Food and Drug Administration (FDA) for AD targeting aβ have documented serious adverse effects like brain swelling (Mahase, 2021) or intracerebral hemorrhage (van Dyck et al., 2023) in clinical trials, there is a heightened level of concern. Among subjects in the early stages of AD, the use of gantenerumab led to a decrease in amyloid plaque buildup when compared to a placebo, but no apparent link was observed between the use of the said antibody treatment and a slowdown in the progression of clinical deterioration (Bateman et al., 2023). Whether inhibiting amyloid plaque alone is enough to prevent the aging brain from cognitive decline or we have underestimated how human brains becoming vulnerable to hemorrhage (Couzin-Frankel and Piller, 2022), hemoglobin change (Ferrer et al., 2011; Min and Min, 2016; Dos Santos and Pardi, 2020; Arioz et al., 2021), hemolytic anemia (Klei et al., 2019), and/or altered hematopoiesis (Aman, 2023; Sanchez Vela et al., 2023) in the progression of aging remains to be resolved. Varying perspectives have risen on the origins of AD. It has been argued that plaques or tangles serve as the fundamental cause, while other perception highlights that aβ or tau are manifestations rather than triggers (Braak and Braak, 1997; Yildirim Simsir et al., 2018). The primary indicator of the ailment is identified as glucose hypometabolism (Mosconi et al., 2008; Tondo et al., 2020), providing a more reliable predictor of cognitive decline than the buildup of plaques or tau. Recognizing that metabolic anomalies in the brain precede Alzheimer’s helps in comprehending why individuals may possess amyloid plaques without developing the disease (Yildirim Simsir et al., 2018).

The failure of antibodies targeting amyloid plaques to prevent cognitive decline in individuals treated during the early stages of AD reported in the prior clinical trial (Bateman et al., 2023) underscores the existence of a distinct mechanism that contributes to the deterioration of cognitive function as individuals age. In addition to the observed deficit in DCX, our data indicates the need for addressing neuroaxonal injury related to tubulin in the caudate nucleus affected by AD. DCX binds to and stabilizes microtubules, which are structural components of the neuronal cytoskeleton. Beta-tubulins are integral members of the tubulin protein family, responsible for the formation and organization of microtubules. Our findings substantiate the connection between deficient DCX and the depletion of neurite components (TUBB, TUBB3, and TUBBP1), representing different forms of beta-tubulin proteins (Supplementary Figures S3, S4). Given that doublecortin (DCX) also facilitates plasticity and learning (Vukovic et al., 2013; Chen et al., 2016; Jalayeri-Darbandi et al., 2018), the rectification of inadequate DCX levels in the basal ganglia affected by AD is justified.

What is the underlying factor responsible for alterations in markers associated with glucose dysmetabolism (GLP-1R), dyslipidemia (LDLRAP1), ferroptosis (NFE2L2), and autophagy (SQSTM1)? It might be lifestyle or diet. Many developed countries worldwide promote polyunsaturated fatty acid (PUFA) as part of a healthy diet by branding “seed” into “vegetable” oils. As a result, consumption of saturated fats from animals has steadily decreased while PUFAs from plants have drastically escalated. Given the Minnesota survey conducted during year 1968–1973, without Ramsden (Ramsden et al., 2016), the risk of low fat diet would have been buried (Keys, 1961; Frantz et al., 1989; Ramsden et al., 2016) for nearly 50 years: “…*the greater degree of cholesterol-lowering was associated with a higher risk of death*…” (Ramsden et al., 2013). This was further supported by recovering the Sydney Diet Heart study, concluding that,”…substituting dietary linoleic acid in place of saturated fats increased the rates of death from all causes…” (Ramsden et al., 2013).

Unlike recent reports on “*iron overload*” where intraventricular hemoglobin or iron induces hydrocephalus (Strahle et al., 2014, 2021), our findings indicate that there is a disturbance in iron homeostasis, specifically involving iron deficiency, in the disease. Correcting the abnormal expressions of hemoglobin subunit proteins in the brain is proposed as a strategy to prevent or delay motor symptoms (gait disturbance) and cognitive impairment associated with CH in the elderly.

Our prior studies on two factors of proximity to telomeres and high A + T content associated with genetics and epigenetics of human diseases (Lucas et al., 2021; McKnight et al., 2021; Raines et al., 2022; White et al., 2022; Hart et al., 2023; McKnight et al., 2023) suggest that G protein coupled receptors harbor a positive correlation with the full-length nucleotide size (Raines et al., 2022). The result presented herein regarding two factors associated with high mutation rates in mice, rats, chimpanzees, and humans indicates that GLP-1R is an exception during evolution across mice (1,480 bp, 44%) and chimpanzees (16,610 bp, 57%), i.e., A + T contents of GLP-1R in four species are less than 59%, the average of human chromosomes. Even if chimpanzee GLP-1R RNA has exceptionally evolved with the longest nucleotide size (Supplementary Table S2), the relative mutability of GLP-1R is moderate (one of the two factors, not both, satisfied), only affected by proximity to telomeres alone (45 Mb < 50 Mb), as compared to the trend of 143 druggable GPCRs (Raines et al., 2022).

Nuclear factor erythroid 2-related factor 2 (NFE2L2), also known as nuclear factor erythroid-derived 2-like 2, is a transcription factor that in humans is encoded by the NFE2L2 gene, which marks ferroptosis (Luo et al., 2022; Ye et al., 2022; Lin et al., 2023) and autophagy (Pajares et al., 2018; Deng et al., 2020; Xu et al., 2020; Bhattacharjee et al., 2022). Ferroptosis is a type of controlled cell death marked by the iron-dependent buildup of lipid peroxides. In contrast to other forms of cell death like apoptosis or necrosis, ferroptosis entails the deadly accumulation of reactive oxygen species (ROS) and lipid peroxidation, particularly in cell membranes (Jakaria et al., 2021; Yadav et al., 2023). Sequestosome-1, encoded by the SQSTM1 gene in humans and commonly referred to as the ubiquitin-binding protein p62, serves as an autophagosome cargo protein. SQSTM1 plays a role in selective autophagy (Tian et al., 2013), which is the natural, conserved degradation of the cell that removes unnecessary or dysfunctional components through a lysosome-dependent regulated mechanism. Other than cancer cells or certain types of epithelial cells (Gupta et al., 2023), emerging research suggests that neurons can undergo ferroptosis and autophagy (Dong et al., 2022; Duan et al., 2023) under certain conditions (Liang et al., 2022; Yang et al., 2023). Ferroptosis and autophagy in neurons have been implicated in various neurodegenerative diseases, including AD. Neuronal populations in the caudate nucleus collectively contribute to cognitive functions within the broader neural circuits involved in cognition and behavior. However, the specific involvement of ferroptosis and autophagy and the markers like NFE2L2 with heme oxygenase 1 (Hmox1) (Zheng et al., 2023) and SQSTM1 in the caudate nucleus has not been extensively studied.

In conclusion, GLP-1 is one of the incretin peptides (Baggio and Drucker, 2007), which significantly modified biology and clinical impact of the gut-pancreas crosstalk from the intestinal mucosa (Baggio and Drucker, 2007). With recent success on the market, there is no doubt that GLP-1R agonist may soon significantly modify diabetes and obesity (Wilkinson et al., 2023; Wolffenbuttel et al., 2023; Yamada et al., 2023). The findings presented in this study suggest that reduced GLP-1R availability in the caudate nucleus in combination with elevated LDLRAP1, might be specific biomarkers of AD.

## Methods

### Human postmortem tissues

Postmortem tissues were requested from the National Institute of Health (NIH) NeuroBioBank (NBB), USA over a period of 1 year. We collected the postmortem tissues of aged individuals through multiple repositories of the NIH NBB, which provided the caudate nucleus (DeVito et al., 2007; Deshpande et al., 2009; Jang et al., 2017; Peterson et al., 2019; Figure 1A) in a frozen state. Caudate nucleus specimens in frozen state were transported to our lab. Per the record provided by the NBB, the specimens were collected at postmortem intervals of 16 ± 8 h (mean ± std.; range 4–25 h after death, *n* = 7 in unaffected controls; n = 7 in NPH; *n* = 5 in AD). Inclusion criteria and diagnosis are provided in Supplementary Tables S3, S4. Seven male and twelve female brain specimens are used, where sex is noted in Supplementary Figure S1 and Supplementary Table S3.

### Data sorting for whole transcriptome RNA-seq

We conducted two different sessions of whole transcriptome RNA-Seq, designed to obtain a total of 62,704 readings (# of genetic loci or genes) with the sample size at N = 16 (*n* = 5 for control and CH; *n* = 6 for AD). The first session involves *N* = 7 (*n* = 2 for control and CH; *n* = 3 for AD). Of all data points (62,704 loci), 3.4% (*n* = 2,144 genetic loci or genes out of 62,704) showed a statistical significance at *p* < 0.05. As these data were sorted per (1) *p*-value, and (2) effect size, one of genes encoding hemoglobin subunit proteins was ranked #1 by statistical significance (*p* = 0.000000000101). The second session was conducted with *N* = 9 (*n* = 3 per group). Of all data points (62,704 loci), 10.8% (*n* = 6,799 genetic loci or genes out of 62,704) showed a statistical significance at *p* < 0.05. Furthermore, 4.8% (*n* = 2,988 genetic loci or genes among 62,704) exhibited a statistical significance at *p* < 0.01. When these data were sorted per (1) *p*-value, and (2) effect size, again, genes encoding hemoglobin subunit proteins were ranked at top by statistical significance along with molecules mediating glucose and lipid metabolisms.

### Primer design

We designed the primers for six genes of interest with one housekeeping gene based on the prior reports (Giaccone et al., 2010; Trabzuni et al., 2012; Mesitskaya et al., 2018; Shim and Madsen, 2018; Gable et al., 2019; Cacabelos, 2020; Hochstetler et al., 2020). Human gene transcripts were searched using Ensembl database.^1^ Using Primer3 online, we determined the sequences of a specific exon per gene transcript.^2^ Then, lyophilized forms were manufactured and provided by the vendor (Thermofisher scientific, Waltham, MA). Seven human gene primers were designed (Supplementary Table S4).

### Total RNA isolation and cDNA generation

Total RNA was extracted from the caudate nucleus specimens of unaffected controls, CH in the elderly cases, and AD cases using the QIA-ZOL-based RNA isolation kit (RNeasy Lipid Tissue Mini Kit, QIAGEN). The concentration and quality of the samples were assessed using a NanoDrop spectrophotometer (Thermofisher). Subsequently, a total of 500 ng of RNA per reaction was reverse-transcribed using the High-Capacity RNA-to-cDNA Kit (Thermofisher; Catalog number: 4368814) with the ABI SimpliAmp Thermal Cycler System (Thermofisher).

### Reverse transcription polymerase chain reaction

RT-PCR was conducted in 25 μL reaction volumes containing 250 ng cDNA, following the manufacturer’s instructions (GoTaq^®^ Green Master Mix). The cycling conditions comprised three steps: denaturation at 95°C for 2 min, followed by 35 cycles of denaturation at 95°C for 30 s, annealing at 60°C for 30 s, and extension at 72°C for 30 s (Promega, Madison, WI). Subsequently, the PCR products were separated through electrophoresis on 1.25% agarose gels in 1x Tris/boric acid/EDTA (TBE) buffer and visualized by staining with Maestro dye (MaestroSafe, Maestrogen). The fluorescent signal was visualized using FluorChem E System (biotechne).

### ImageJ analysis

The analysis of DNA agarose gel images was performed using NIH ImageJ. The procedure consists of six steps: (1) Open the gel image in ImageJ, (2) Use the rectangle tool to select, (3) Analyze-gels-1st lane and subsequent lanes until the end, (4) Analyze-gels-plot lanes, (5) Connect with straight lines, and (6) Select with points. The area calculated for each band was recorded in the result file and saved in a spreadsheet. The relative fold change for each gene of interest was quantified relative to the expression of the housekeeping gene.

### Statistical analysis

Primary component analysis (PCA) and statistical analyses were carried out using Prism (version 9.3.0, GraphPad Software Inc.), allowing for the creation of a heatmap plot and bar graphs based on the data analyzed with ImageJ. Non-parametric tests were employed for their conservative approach compared to parametric tests. Consequently, the Mann–Whitney test and Kruskal–Wallis test were utilized for two-group and three-group comparisons, respectively. Statistical significance was considered when the *p*-value was less than 0.05, and significance levels are denoted in the figures and legends as *p < 0.05, **p < 0.01, and ***p < 0.005.

### Gene set enrichment analysis and hierarchical clustering

GSEA 4.3.3 and G-profiler were used for gene set enrichment analysis (GSEA). For hierarchical clustering of the RNA-Seq dataset, providing dendrograms, Instant Clue software was used.

## Data availability statement

The original contributions presented in the study are included in the article/Supplementary material, further inquiries can be directed to the corresponding author/s.

## Ethics statement

The requirement of ethical approval was waived by the Marshall University Research Corporation for the studies on humans because it is the human postmortem sample. The studies were conducted in accordance with the local legislation and institutional requirements. Written informed consent for participation was not required from the participants or the participants’ legal guardians/next of kin in accordance with the national legislation and institutional requirements. The human samples used in this study were acquired from the National Institute of Health NeuroBioBank.

## Author contributions

EB: Data curation, Formal analysis, Investigation, Validation, Writing – original draft, Writing – review & editing. GI: Data curation, Formal analysis, Investigation, Writing – review & editing. AC: Investigation, Validation, Writing – review & editing. GC: Investigation, Validation, Writing – review & editing. TP: Investigation, Validation, Writing – review & editing. CL: Validation, Writing – review & editing. PP: Project administration, Resources, Writing – review & editing. JBD: Writing – review & editing. PL: Writing – review & editing. JS: Conceptualization, Formal analysis, Funding acquisition, Project administration, Supervision, Writing – original draft.

## Footnotes

^1^http://useast.ensembl.org/index.html

^2^https://bioinfo.ut.ee/primer3-0.4.0/
