# Supplementary material for: Reduced GLP-1R availability in the caudate nucleus with Alzheimer’s disease
Source: Front Aging Neurosci. 2024 Jun 10;16:1350239. doi: 10.3389/fnagi.2024.1350239 (PMC11194438; doi:10.3389/fnagi.2024.1350239)
Supplement: Supplementary file 1 [file Data_Sheet_1.PDF]

Supplementary Material for

Reduced GLP-1R availability in the caudate nucleus with Alzheimer's disease

Emma Barrett[1], Gabrielle Ivey[1], Adam Cunningham[1], Gary Coffman[1], Tyera Pemberton[1], Chan Lee[2], Prabir Patra[1], Peter H.U. Lee[3,4], Joon W. Shim[1*]

Correspondence to: shim@marshall.edu

This file includes:

Supplementary methods,
Figure S1 to S14,
Tables S1 to S5

Supplementary methods:

Cell culture

The human endothelial cells (EA.hy026, ATCC) were purchased and received in a frozen state. To thaw the frozen cells, they were placed at 37°C for two minutes. The cell suspension (1 mL) was then resuspended with 9 mL of fresh low glucose Dulbecco's Modified Eagle's Medium (DMEM: Gibco; Carlsbad, CA) in a cryogenic vial. This solution contained 10% fetal bovine serum (FBS; BD Biosciences; San Jose, CA) and 1% 1× antibiotic-antimycotic (ABAM) which was then preheated at 37°C. The cells were then placed in a Falcon ® flask (Thermo Scientific, Waltham MA) where approximately 50% of them were attached to the plate in 2 hours. It was observed that more than 90% of the suspended cells were attached to the bottom of the flask the following day. The cells displayed growth without medium change for the next week. The cells were counted using a hemacytometer which calculated a total of 5 x 10^5 cells/mL. These cells were divided into a 6-well plate pre-incubated. A coverslip was placed at the bottom of each well which marked day 1 of the experimental period. At this stage, the cells were cultured with fresh, new medium for two weeks; the cells on each coverslip were harvested on days 3, 7, 10, and 14 for immunocytochemistry.

Animal procedures

The C57BL6 mice were bred as described previously. Adult mice (age 12 to 20 months), both male and female were randomly assigned to be used in histology. At the time of sacrifice, animals were anesthetized with an overdose of isoflurane (3.5% nasal). The body was flushed with saline and perfused intracardially with 4% paraformaldehyde (PFA).

Histology

Formalin fixed human caudate nucleus specimens were obtained from the NIH Neurobiobank. Harvest mouse brains and guts (stomach, large and small intestine) with human postmortem specimens were immersed in 30% sucrose in PBS at 4 °C for 2-3 days or when cryo-protection is completely carried out. The tissue in sucrose solution was briefly washed with PBS and further placed in a square mold with optimal cutting temperature (O.C.T.) compound (Tissue-Tek) at

−170 °C using dry ice and isopentane (Fisher Scientific), in a rectangular aluminum tray. Snap-frozen molds containing tissue specimen were kept at −80 °C until cryo-sectioning.

Immunohistochemistry

Primary antibodies were the following: rabbit anti-αSMA (1:100, Sigma) and rabbit anti- elastin (1:200, Abcam). For DAB substrate, ABC kit (VectorLabs) was used. Secondary antibodies were goat anti-rabbit IgG (diluted 1:200 in blocking solution). Bright-field micrographic images were taken on Keyence microscope (Osaka, Japan/the United State branch in Boston MA).

Distance to a telomere and nucleotide composition calculation

To determine the distance from the gene of interest to its telomere and calculate adenine and thymine (A + T) content percentage of nucleotides, we used the NCBI Genome Data Viewer (https://www.ncbi.nlm.nih.gov/genome/gdv/) and the publicly available GC Content Calculator (https://www.biologicscorp.com/tools/GCContent/#.XvctCi-z2uV). This allowed us to access the adenine and thymine in percent along with the full-length base-pair sizes of the nucleotide (Lucas et al., 2021;McKnight et al., 2021;Raines et al., 2022).

Measuring the distance or the proximity to telomeres

The biological basis for a high mutation rate in human chromosomes was previously described by Nusbaum and colleagues (Nusbaum et al., 2006). have described the biological basis for the high mutation rate in human chromosomes. In this study, we have adopted this established method based on their suggestion (Nusbaum et al., 2006). We have adopted one of the three factors associated with high mutation rates (Nusbaum et al., 2006) to determine the proximity of a gene to its telomere (Lucas et al., 2021;McKnight et al., 2021;Raines et al., 2022). We have identified the location of seven genes in mouse, rat, and human chromosomes to approximate each of the gene's A + T content as well as the location of their corresponding telomere with the premise below:

(1) If recombination frequency is shorter than (≤) 50 centimorgan (cM), then the genes are linked.

(2) If recombination frequency is longer than 50 cM, then the genes are not linked.

For the previously mentioned measurement of cM: 1 cM ≅ 1 million bases (Mb) (Hastbacka et al., 1992)

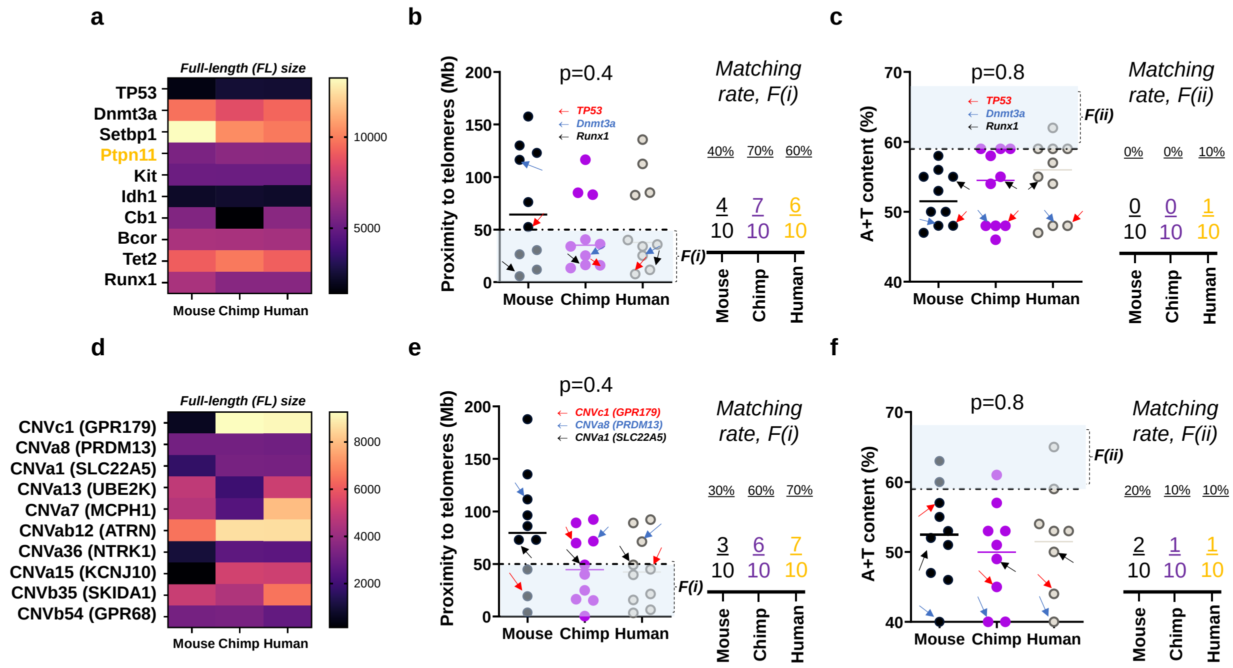

Figure S1. Relative mutability of hematopoiesis-driver genes and copy number variation loci mimicking Space Missions (a) The full-length (FL) size of ten genes reported to drive hematopoiesis after a short-term Space Missions over three species. (b) Proximity to telomeres of ten genes over three species. Note that six of ten human genes investigated in this study have evolved in a way meeting proximity to telomeres or the first factor, F(i), associated with high mutation rate as 60% genes are located at less than 50 Mb as compared to those of mice. (c) A+T content of ten genes over three species. Nine of ten human genes demonstrate a similar characteristic of difficulty in meeting this second factor, F(ii), associated with high mutation rate such as TP53. Arrows in red, blue, and black indicating TP53, Dnmt3a, and Runx1, respectively (a-c). (d) The FL size of ten genes reported to show copy number variation (CNV) mutations after ionizing radiation mimicking Space Missions over three species. (e) Proximity to telomeres of ten genes over three species. Note that six of ten human genes investigated in this study have evolved in a way meeting proximity to telomeres or the first factor, F(i), associated with high mutation rate as 60% genes are located at less than 50 Mb as compared to those of mice. (f) A+T content of ten genes over three species. Nine of ten human genes demonstrate a similar characteristic of difficulty in meeting this second factor, F(ii), associated with high mutation rate such as TP53. Arrows in red, blue, and black indicating TP53, Dnmt3a, and Runx1, respectively (a-c).

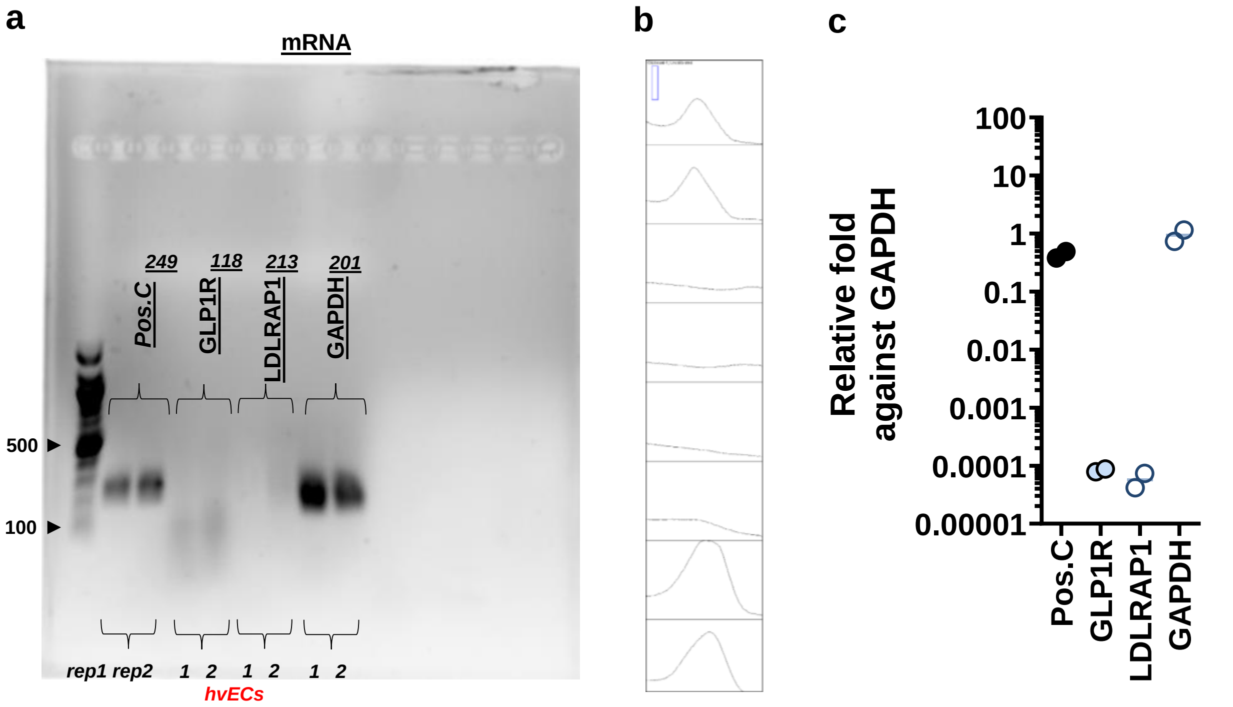

Figure S2. Identification of human GLP1R and LDLRAP1 transcript in endothelial cells *in vitro* (a) Agarose gel exhibiting mRNA expressions of GLP1R, LDLRAP1, and GAPDH. Pos.C, positive control with the known nucleotide size at 249 bases. Rep., replicate; hvECs, human vascular endothelial cells. (b) Quantification of DNA bands shown in the DNA gel (a) using NIH ImageJ. (c) Scatter plot summarizing data shown in a-b. Note that the relative levels of GLP1R and LDLRAP1 mRNA are almost near at zero, while the control and GAPDH mRNA are at 1-fold, as compared to the internal reference gene (GAPDH).

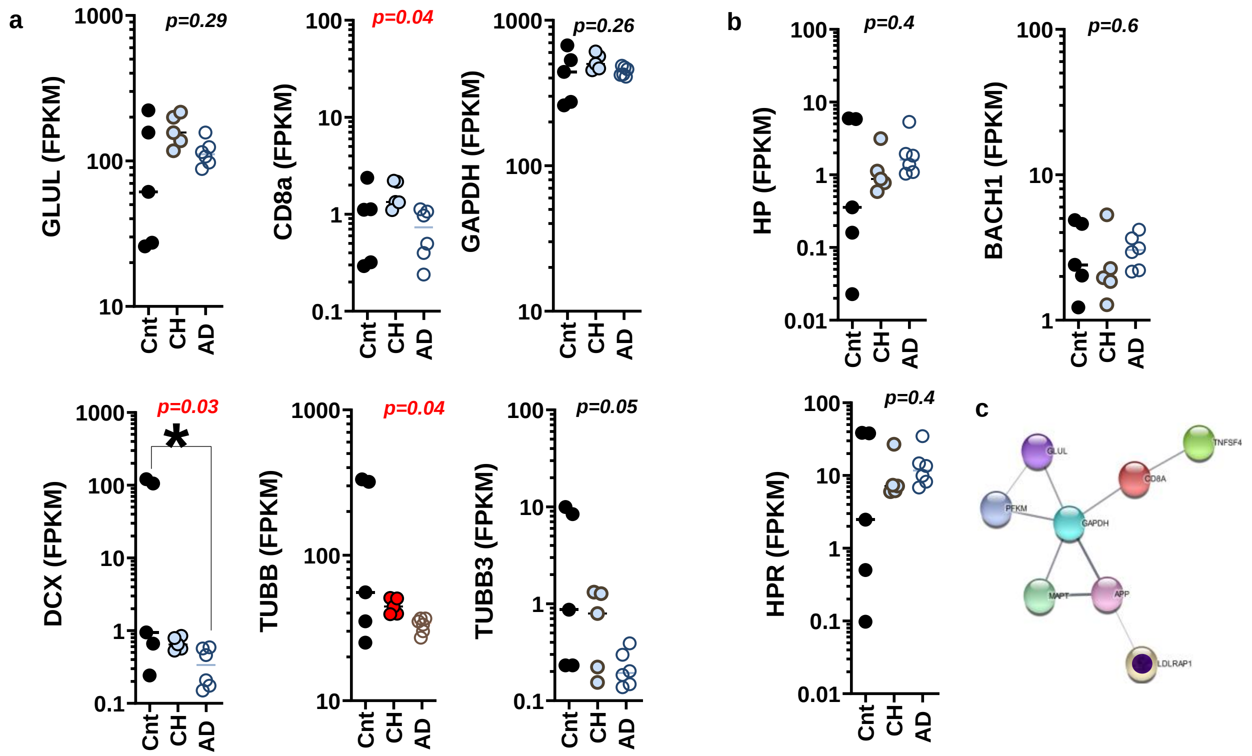

Figure S3. Transcript levels of genes mediating glucose metabolism, inflammation, and axonal injury in the aged brain (a) the scatter plots summarizing mRNA levels of GLUL and GAPDH that mediate glucose metabolism, CD8a (a marker for infiltration of peripheral inflammatory cells into the CNS), DCX (a marker for young neuron), TUBB (a marker for tubulin or microtubule), and TUBB3 (a marker for Tuj1 or mature neuron) in the caudate nucleus with CH and AD as compared to those of Cnt obtained from the whole transcriptome RNA-Seq; Cnt, control; CH, chronic hydrocephalus; AD, Alzheimer's disease; statistical analysis by Kruskall Wallis test. (b) Genes mediating autophagy (BACH1) and hemolytic anemia associated with the main figure (Fig. 3) (c) a network chart showing interconnections and association among LDLRAP1, and seven other genes depicted in (a) and the main figure (Fig. 2). A putative core gene for AD marked with an inner circle in purple (c).

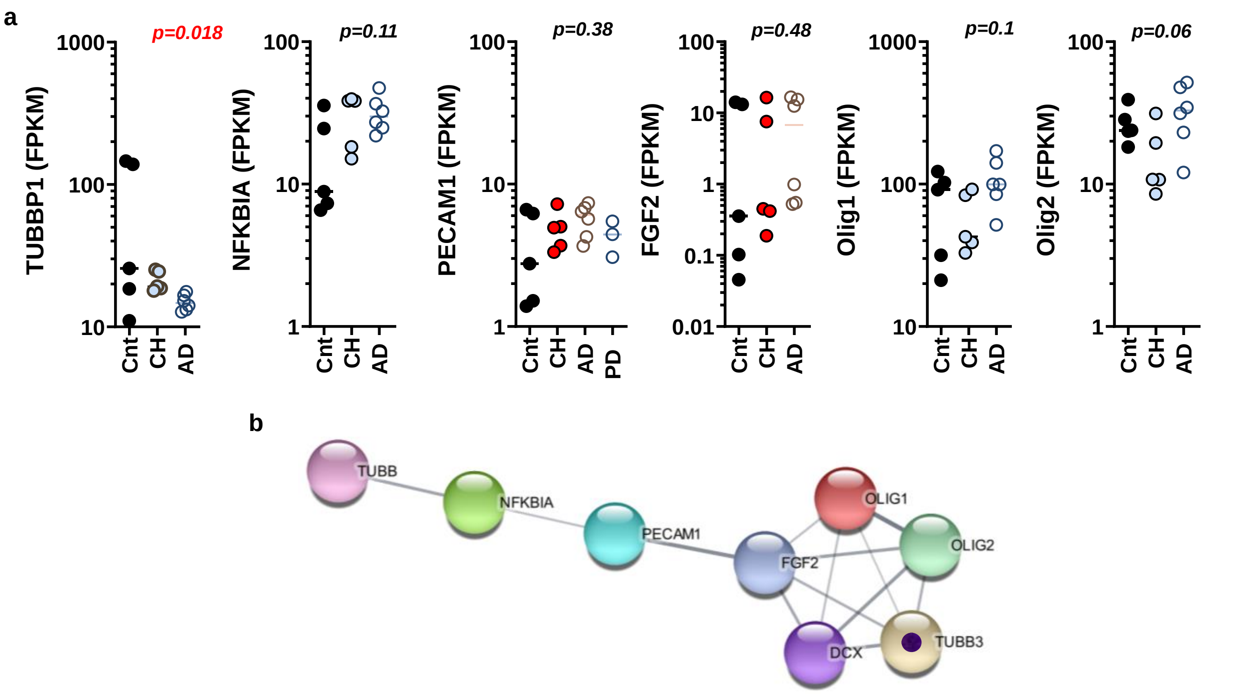

Figure S4. Transcript levels of genes related to beta-tubulin, oligodendrocytes, and angiogenesis in the aged brain (a) the scatter plots summarizing mRNA levels of TUBBP1, NFKBIA, PECAM1, FGF2, OLIG1, and OLIG2 in the caudate nucleus with CH and AD as compared to those of Cnt obtained from the whole transcriptome RNA-Seq; statistical analysis by Kruskall Wallis test. (b) a network chart showing interconnections and association between TUBBP1 reported to interact with TUBB and seven other genes shown in (a) and Fig. S2. A putative core gene for AD marked with an inner circle in purple (b).

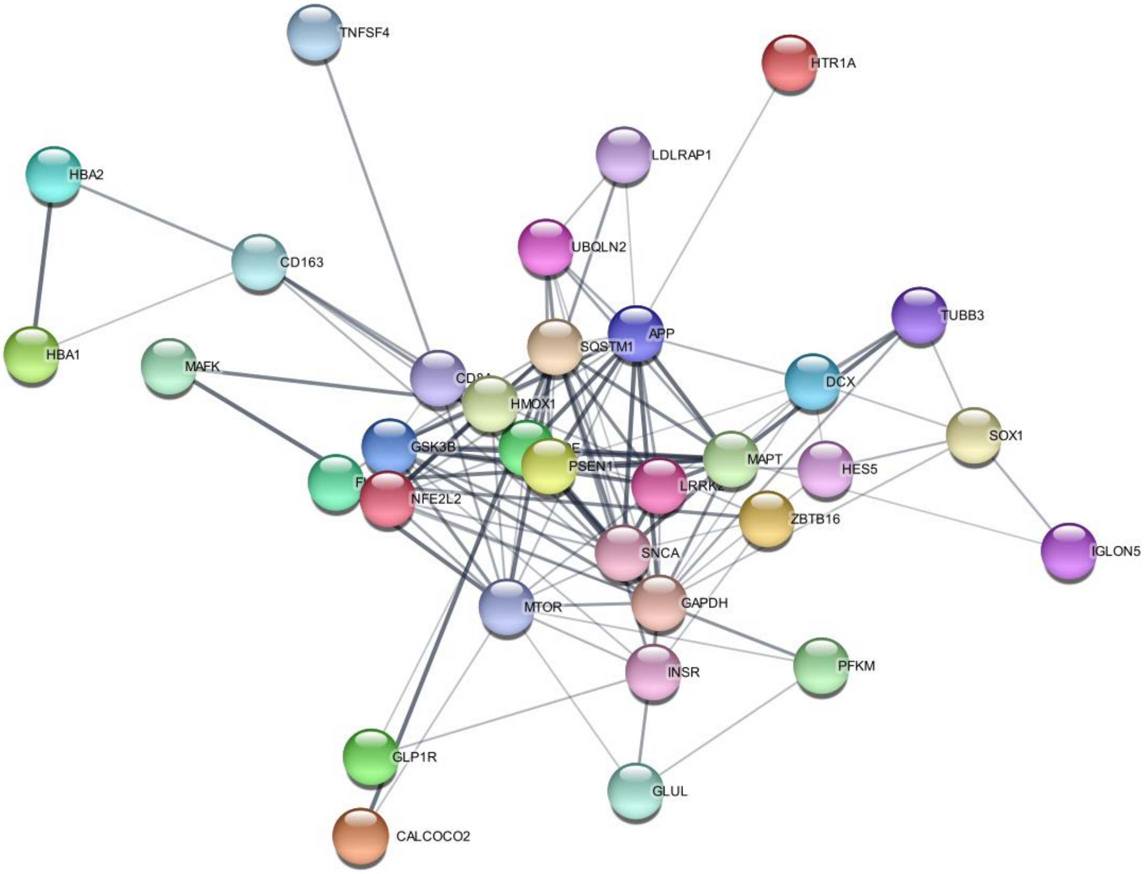

Figure S5. Comprehensive networks of genes encoding GLP-1R related clusters in part shown in Figs. 1-4: separate gene networks shown in Fig. 1-4 were connected as ubiquitous or housekeeping molecule like GAPDH is added as a linker.

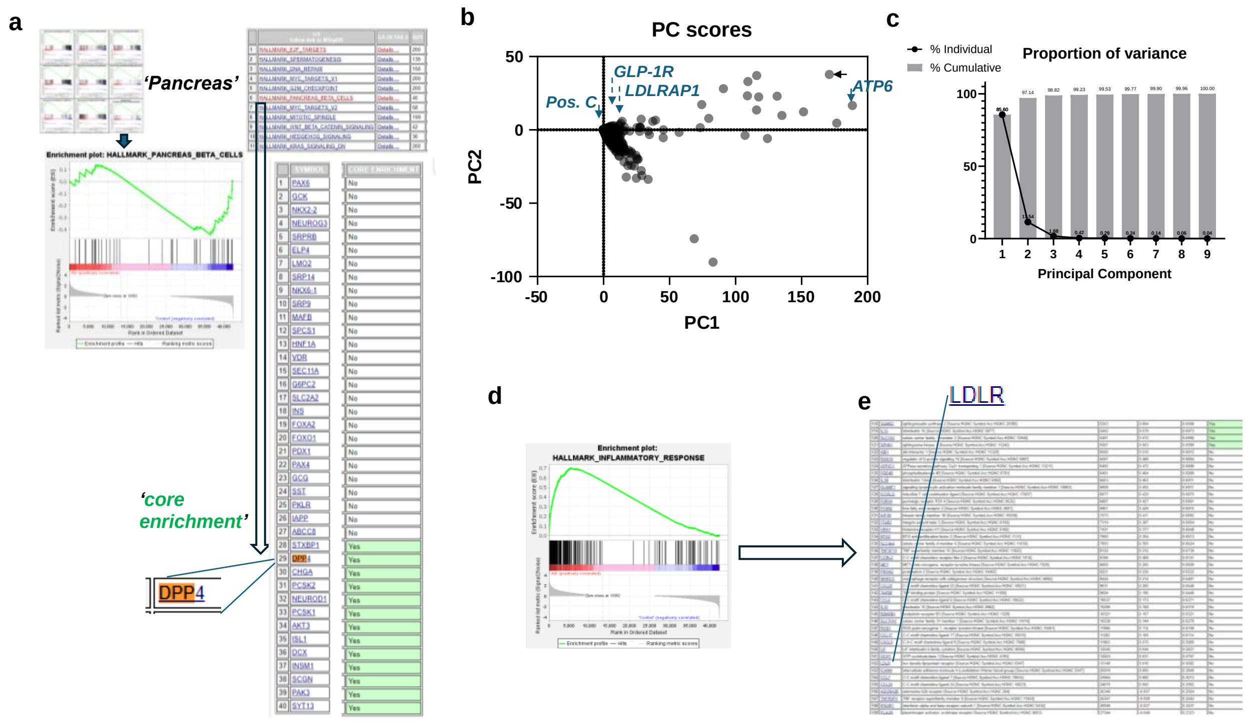

Figure S6. Gene set enrichment analysis (GSEA) of RNA-Seq data suggesting incretin (a) GSEA analysis suggesting 'pancreas' beta cells (arrowhead) and gene set members involving incretin such as DPP-4 (arrow) as part of a 'core enrichment' set (columns in green marked with 'Yes') (b) PCA analysis showing more than two times wider variations spanning Pos. C at 0 to ATP6 at 200 along primary component 1 (PC1) than PC2 (roughly -100 to 50; arrows in black). GLP-1R at approximately 5 (rank order 2,431) and LDLRAP1 near at 10 (rank order 3,815) when projected to the axis of PC1 (dotted arrows in blue); Pos. C, positive control; ATP6, mitochondrially encoded ATP synthase membrane subunit 6. (c) Bar graph showing PC1 and PC2 representing 97.14% of variances found in the RNA-Seq dataset when comparing control and AD group (d) Enrichment plot from GSEA analysis showing enrichment score vs. rank in ordered dataset comparing control vs. AD. (e) GSEA-driven list of gene sets enriched in phenotype AD related to inflammatory response containing LDLR (arrow) forming a basis to connect with LDLRAP1.

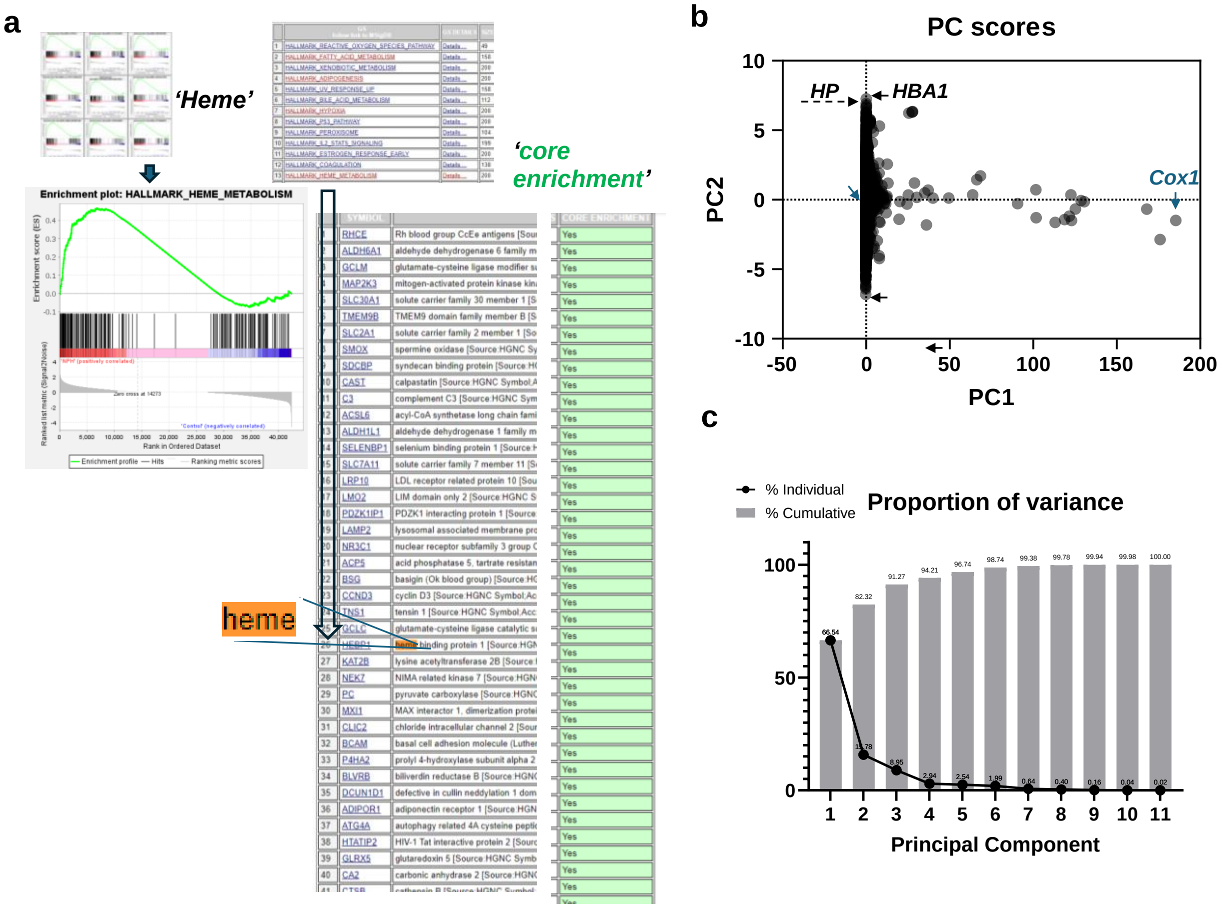

Figure S7. Gene set enrichment analysis (GSEA) of RNA-Seq data on CH suggesting heme (a) GSEA analysis suggesting hemoglobin or heme (arrowhead) and gene set members involving heme (arrow) as part of a 'core enrichment' set (columns in green marked with 'Yes') (b) PCA analysis showing more than ten times wider variations spanning 0 to 200 along primary component 1 (PC1) than PC2 (roughly -10 to 10; arrows in black). HBA1 at 7.25 (rank order 10) and HP near at 6.7 (rank order 1) when projected to the axis of PC2 (dotted arrows in black); HP, haptoglobin; HBA1, hemoglobin subunit protein 1; Cox1, cytochrome c oxidase subunit 1. (c) Bar graph showing PC1 and PC2 representing 82% of variances found in the RNA-Seq dataset when comparing control and CH group

a

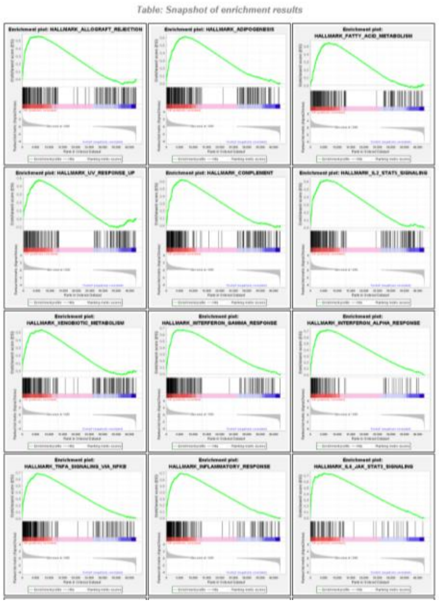

b

	GS follow link to MSigDB	GS DETAILS	SIZE	ES	NES	NOM p-val	FDR q-val	FWER p-val	RANK AT MAX	LEADING EDGE
1	HALLMARK_ALLOGRAFT_REJECTION	Details ...	200	0.56	1.49	0.000	0.508	0.182	6060	tags=44%, list=14%, signal=51%
2	HALLMARK_ADIPOGENESIS	Details ...	200	0.62	1.48	0.000	0.282	0.237	6891	tags=51%, list=16%, signal=60%
3	HALLMARK_FATTY_ACID_METABOLISM	Details ...	158	0.55	1.47	0.000	0.206	0.237	6855	tags=47%, list=16%, signal=56%
4	HALLMARK_UV_RESPONSE_UP	Details ...	158	0.48	1.47	0.000	0.168	0.237	5737	tags=39%, list=14%, signal=44%
5	HALLMARK_COMPLEMENT	Details ...	200	0.62	1.45	0.000	0.192	0.344	5816	tags=54%, list=14%, signal=62%
6	HALLMARK_IL2_STAT5_SIGNALING	Details ...	199	0.62	1.45	0.000	0.178	0.344	6257	tags=51%, list=15%, signal=59%
7	HALLMARK_XENOBIOTIC_METABOLISM	Details ...	200	0.53	1.45	0.000	0.188	0.394	6855	tags=46%, list=16%, signal=55%
8	HALLMARK_INTERFERON_GAMMA_RESPONSE	Details ...	200	0.67	1.44	0.000	0.171	0.394	4377	tags=49%, list=10%, signal=54%
9	HALLMARK_INTERFERON_ALPHA_RESPONSE	Details ...	97	0.72	1.43	0.000	0.180	0.394	6167	tags=64%, list=15%, signal=75%
10	HALLMARK_TNFA_SIGNALING_VIA_NFKB	Details ...	200	0.69	1.42	0.000	0.171	0.394	5587	tags=58%, list=13%, signal=66%
11	HALLMARK_INFLAMMATORY_RESPONSE	Details ...	200	0.70	1.42	0.000	0.169	0.394	5587	tags=61%, list=13%, signal=69%
12	HALLMARK_IL6_JAK_STAT3_SIGNALING	Details ...	87	0.74	1.41	0.000	0.164	0.394	5245	tags=55%, list=12%, signal=63%
13	HALLMARK_KRAS_SIGNALING_UP	Details ...	200	0.57	1.41	0.111	0.159	0.394	5103	tags=42%, list=12%, signal=47%
14	HALLMARK_APOPTOSIS	Details ...	161	0.58	1.39	0.000	0.158	0.394	5594	tags=49%, list=13%, signal=56%
15	HALLMARK_P53_PATHWAY	Details ...	200	0.55	1.39	0.000	0.151	0.394	6464	tags=49%, list=15%, signal=58%
16	HALLMARK_PI3K_AKT_MTOR_SIGNALING	Details ...	105	0.44	1.36	0.000	0.169	0.496	6076	tags=34%, list=14%, signal=40%
17	HALLMARK_COAGULATION	Details ...	138	0.57	1.36	0.000	0.162	0.496	7443	tags=47%, list=18%, signal=57%
18	HALLMARK_PROTEIN_SECRETION	Details ...	96	0.58	1.35	0.000	0.156	0.496	7477	tags=50%, list=18%, signal=61%
19	HALLMARK_HYPOXIA	Details ...	200	0.56	1.34	0.000	0.153	0.496	5304	tags=42%, list=13%, signal=47%
20	HALLMARK_PEROXISOME	Details ...	104	0.48	1.34	0.000	0.159	0.496	6055	tags=38%, list=14%, signal=45%
21	HALLMARK_BILE_ACID_METABOLISM		112	0.57	1.32	0.000	0.170	0.548	7218	tags=52%, list=17%, signal=62%
22	HALLMARK_CHOLESTEROL_HOMEOSTASIS		74	0.51	1.31	0.195	0.178	0.548	7129	tags=53%, list=17%, signal=63%
23	HALLMARK_MYOGENESIS		200	0.48	1.30	0.000	0.184	0.650	7507	tags=48%, list=18%, signal=58%
24	HALLMARK_HEME_METABOLISM		200	0.45	1.29	0.000	0.182	0.650	6344	tags=42%, list=15%, signal=49%
25	HALLMARK_TGF_BETA_SIGNALING		54	0.60	1.28	0.187	0.193	0.701	6530	tags=46%, list=15%, signal=55%
26	HALLMARK_ESTROGEN_RESPONSE_EARLY		200	0.46	1.25	0.112	0.207	0.741	5733	tags=37%, list=14%, signal=43%
27	HALLMARK_MTORC1_SIGNALING		200	0.48	1.25	0.000	0.201	0.741	7218	tags=47%, list=17%, signal=56%
28	HALLMARK_EPITHELIAL_MESENCHYMAL_TRANSITION		200	0.51	1.21	0.189	0.236	0.786	6349	tags=45%, list=15%, signal=52%
29	HALLMARK_ANDROGEN_RESPONSE		101	0.52	1.20	0.101	0.243	0.826	7246	tags=50%, list=17%, signal=60%
30	HALLMARK_REACTIVE_OXYGEN_SPECIES_PATHWAY		49	0.53	1.20	0.099	0.239	0.826	6500	tags=47%, list=15%, signal=55%
31	HALLMARK_GLYCOLYSIS		200	0.38	1.20	0.110	0.234	0.826	7554	tags=40%, list=18%, signal=48%
32	HALLMARK_ESTROGEN_RESPONSE_LATE		200	0.43	1.18	0.192	0.245	0.826	6805	tags=42%, list=16%, signal=50%
33	HALLMARK_APICAL_JUNCTION		200	0.35	1.16	0.107	0.271	0.826	3890	tags=25%, list=9%, signal=27%
34	HALLMARK_NOTCH_SIGNALING		32	0.49	1.08	0.477	0.346	0.826	6091	tags=47%, list=14%, signal=55%
35	HALLMARK_UNFOLDED_PROTEIN_RESPONSE		113	0.38	0.99	0.581	0.453	0.941	5954	tags=30%, list=14%, signal=35%
36	HALLMARK_ANGIOGENESIS		36	0.40	0.94	0.485	0.516	0.941	6412	tags=36%, list=15%, signal=43%
37	HALLMARK_UV_RESPONSE_DN		144	0.35	0.89	0.684	0.595	0.941	4668	tags=27%, list=11%, signal=30%
38	HALLMARK_APICAL_SURFACE		44	0.30	0.81	0.679	0.728	1.000	7011	tags=34%, list=17%, signal=41%
39	HALLMARK_OXIDATIVE_PHOSPHORYLATION		200	0.37	0.71	0.892	0.880	1.000	8044	tags=31%, list=19%, signal=37%

Figure S8. Enrichment plots when comparing control and AD (a) Multiple gene sets enriched in AD (b) Detailed list of gene sets enriched in AD

a

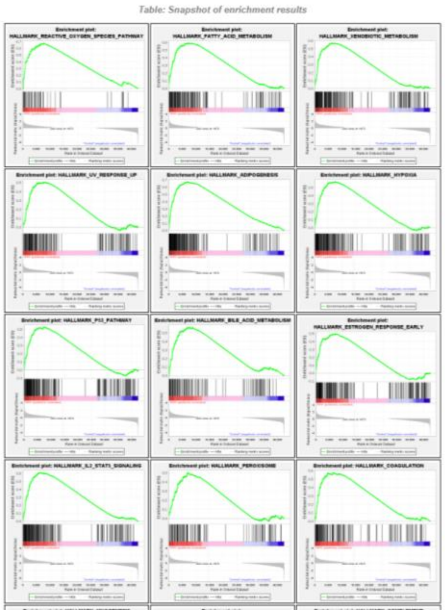

b

	GS follow link to MSigDB	GS DETAILS	SIZE	ES	NES	NOM p-val	FDR q-val	FWER p-val	RANK AT MAX	LEADING EDGE
1	HALLMARK_REACTIVE_OXYGEN_SPECIES_PATHWAY	Details ...	49	0.68	1.48	0.000	1.000	0.329	7627	tags=71%, list=18%, signal=87%
2	HALLMARK_FATTY_ACID_METABOLISM	Details ...	158	0.59	1.46	0.000	0.587	0.382	7197	tags=54%, list=17%, signal=65%
3	HALLMARK_XENOBIOTIC_METABOLISM	Details ...	200	0.57	1.46	0.000	0.409	0.382	6192	tags=48%, list=15%, signal=55%
4	HALLMARK_UV_RESPONSE_UP	Details ...	158	0.51	1.43	0.000	0.383	0.428	7738	tags=50%, list=18%, signal=61%
5	HALLMARK_ADIPOGENESIS	Details ...	200	0.67	1.41	0.000	0.326	0.428	6828	tags=61%, list=16%, signal=72%
6	HALLMARK_HYPOXIA	Details ...	200	0.57	1.40	0.086	0.310	0.428	7678	tags=58%, list=18%, signal=70%
7	HALLMARK_P53_PATHWAY	Details ...	200	0.52	1.39	0.000	0.273	0.428	7717	tags=53%, list=18%, signal=64%
8	HALLMARK_BILE_ACID_METABOLISM	Details ...	112	0.56	1.37	0.000	0.288	0.471	6771	tags=45%, list=16%, signal=53%
9	HALLMARK_ESTROGEN_RESPONSE_EARLY	Details ...	200	0.50	1.34	0.000	0.282	0.522	7031	tags=48%, list=17%, signal=57%
10	HALLMARK_IL2_STAT5_SIGNALING	Details ...	199	0.61	1.34	0.000	0.268	0.571	6000	tags=52%, list=14%, signal=60%
11	HALLMARK_PEROXISOME	Details ...	104	0.50	1.33	0.000	0.249	0.571	6992	tags=46%, list=16%, signal=55%
12	HALLMARK_COAGULATION	Details ...	138	0.58	1.30	0.090	0.260	0.571	6978	tags=46%, list=16%, signal=55%
13	HALLMARK_MYOGENESIS	Details ...	200	0.49	1.29	0.103	0.252	0.623	8254	tags=49%, list=19%, signal=61%
14	HALLMARK_ESTROGEN_RESPONSE_LATE	Details ...	200	0.46	1.29	0.000	0.257	0.623	7544	tags=48%, list=18%, signal=58%
15	HALLMARK_COMPLEMENT	Details ...	200	0.60	1.29	0.000	0.255	0.623	6330	tags=53%, list=15%, signal=62%
16	HALLMARK_HEME_METABOLISM	Details ...	200	0.47	1.28	0.101	0.248	0.623	6876	tags=44%, list=16%, signal=52%
17	HALLMARK_APOPTOSIS	Details ...	161	0.54	1.26	0.193	0.268	0.731	7836	tags=58%, list=18%, signal=71%
18	HALLMARK_OXIDATIVE_PHOSPHORYLATION	Details ...	200	0.71	1.25	0.093	0.259	0.731	9215	tags=80%, list=22%, signal=102%
19	HALLMARK_MTORC1_SIGNALING	Details ...	200	0.50	1.23	0.000	0.296	0.731	8578	tags=58%, list=20%, signal=73%
20	HALLMARK_TNFA_SIGNALING_VIA_NFKB	Details ...	200	0.67	1.22	0.180	0.300	0.731	7808	tags=72%, list=18%, signal=88%
21	HALLMARK_INFLAMMATORY_RESPONSE		200	0.67	1.21	0.000	0.296	0.731	7550	tags=67%, list=18%, signal=81%
22	HALLMARK_PI3K_AKT_MTOR_SIGNALING		105	0.46	1.21	0.000	0.285	0.731	7680	tags=46%, list=18%, signal=56%
23	HALLMARK_TGF_BETA_SIGNALING		54	0.52	1.21	0.286	0.287	0.731	6994	tags=46%, list=17%, signal=55%
24	HALLMARK_GLYCOLYSIS		200	0.43	1.20	0.107	0.285	0.731	7678	tags=45%, list=18%, signal=55%
25	HALLMARK_KRAS_SIGNALING_UP		200	0.52	1.19	0.105	0.283	0.731	6075	tags=44%, list=14%, signal=51%
26	HALLMARK_ANDROGEN_RESPONSE		101	0.51	1.18	0.208	0.284	0.731	5874	tags=44%, list=14%, signal=50%
27	HALLMARK_PROTEIN_SECRETION		96	0.52	1.17	0.317	0.285	0.731	7923	tags=48%, list=19%, signal=59%
28	HALLMARK_ALLOGRAFT_REJECTION		200	0.55	1.16	0.196	0.291	0.780	7704	tags=54%, list=18%, signal=66%
29	HALLMARK_IL6_JAK_STAT3_SIGNALING		87	0.72	1.15	0.105	0.291	0.780	7417	tags=69%, list=17%, signal=83%
30	HALLMARK_INTERFERON_GAMMA_RESPONSE		200	0.69	1.12	0.105	0.302	0.780	6070	tags=65%, list=14%, signal=75%
31	HALLMARK_INTERFERON_ALPHA_RESPONSE		97	0.69	1.12	0.291	0.293	0.780	6235	tags=69%, list=15%, signal=81%
32	HALLMARK_CHOLESTEROL_HOMEOSTASIS		74	0.35	1.00	0.323	0.524	0.892	8671	tags=47%, list=20%, signal=59%
33	HALLMARK_UNFOLDED_PROTEIN_RESPONSE		113	0.41	0.99	0.400	0.526	0.892	6256	tags=38%, list=15%, signal=45%
34	HALLMARK_EPITHELIAL_MESENCHYMAL_TRANSITION		200	0.42	0.99	0.470	0.512	0.892	7697	tags=46%, list=18%, signal=55%
35	HALLMARK_KRAS_SIGNALING_DN		200	0.32	0.98	0.389	0.526	0.892	9154	tags=40%, list=22%, signal=50%
36	HALLMARK_NOTCH_SIGNALING		32	0.43	0.94	0.489	0.553	0.940	8488	tags=53%, list=20%, signal=66%
37	HALLMARK_UV_RESPONSE_DN		144	0.33	0.89	0.672	0.670	1.000	2947	tags=22%, list=7%, signal=24%
38	HALLMARK_APICAL_JUNCTION		200	0.27	0.89	0.689	0.653	1.000	5053	tags=25%, list=12%, signal=28%
39	HALLMARK_APICAL_SURFACE		44	0.32	0.83	0.676	0.716	1.000	2315	tags=20%, list=5%, signal=22%
40	HALLMARK_HEDGEHOG_SIGNALING		36	0.35	0.82	0.890	0.714	1.000	7298	tags=44%, list=17%, signal=54%
41	HALLMARK_ANGIOGENESIS		36	0.30	0.77	0.781	0.860	1.000	8316	tags=42%, list=20%, signal=52%

Figure S9. Enrichment plots when comparing control and CH (a) Multiple gene sets enriched in CH (b) Detailed list of gene sets enriched in CH

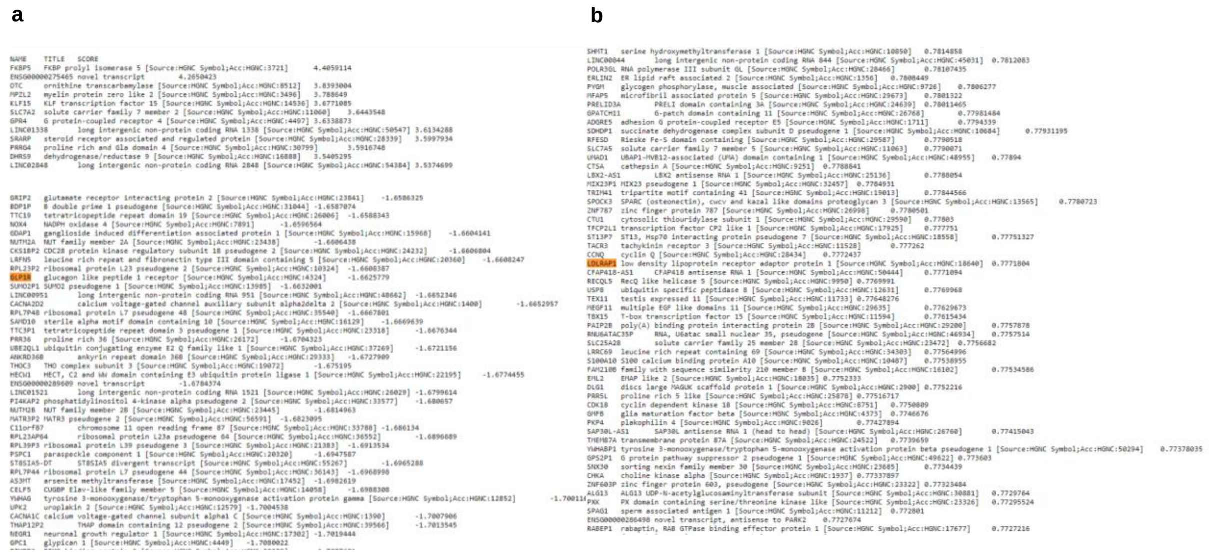

Figure S10. Ranked gene score obtained through GSEA (a) Sets including GLP-1R (highlighted in color) (b) Sets including LDLRAP1 (highlighted in color)

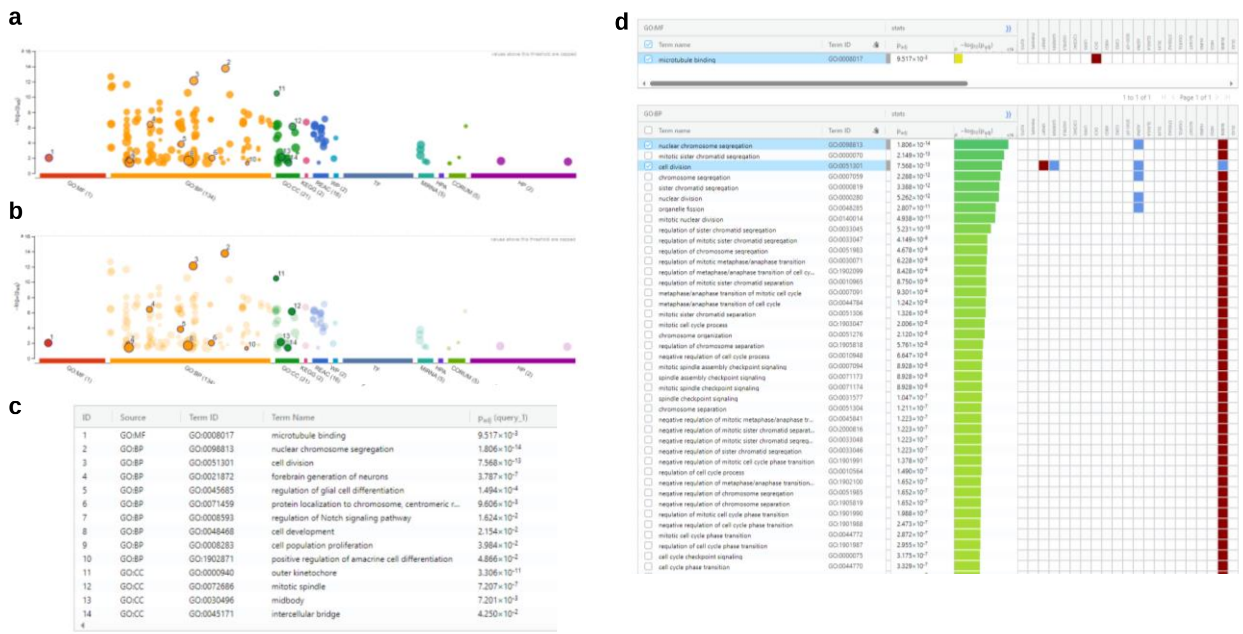

ID	Source	Term ID	Term Name	P_{adj} (query_1)
1	GO:MF	GO:0008017	microtubule binding	9.517×10^{-3}
2	GO:BP	GO:0098813	nuclear chromosome segregation	1.806×10^{-14}
3	GO:BP	GO:0051301	cell division	7.568×10^{-13}
4	GO:BP	GO:0021872	forebrain generation of neurons	3.787×10^{-7}
5	GO:BP	GO:0045685	regulation of glial cell differentiation	1.494×10^{-4}
6	GO:BP	GO:0071459	protein localization to chromosome, centromeric r...	9.606×10^{-3}
7	GO:BP	GO:0008593	regulation of Notch signaling pathway	1.624×10^{-2}
8	GO:BP	GO:0048468	cell development	2.154×10^{-2}
9	GO:BP	GO:0008283	cell population proliferation	3.984×10^{-2}
10	GO:BP	GO:1902871	positive regulation of amacrine cell differentiation	4.866×10^{-2}
11	GO:CC	GO:0000940	outer kinetochore	3.306×10^{-11}
12	GO:CC	GO:0072686	mitotic spindle	7.207×10^{-7}
13	GO:CC	GO:0030496	midbody	7.201×10^{-3}
14	GO:CC	GO:0045171	intercellular bridge	4.250×10^{-2}

Figure S11. Enrichment analysis of AD dataset through G profiler (a) enrichment of select 76 genes from AD dataset (b) enrichment of 14 capped genes among 76 genes from AD dataset (c) 14 capped genes among 76 analyzed: whether the -log10 (p-values) would be capped if >16. (d) Detailed results analyzed by G profiler involving doublecortin (DCX).

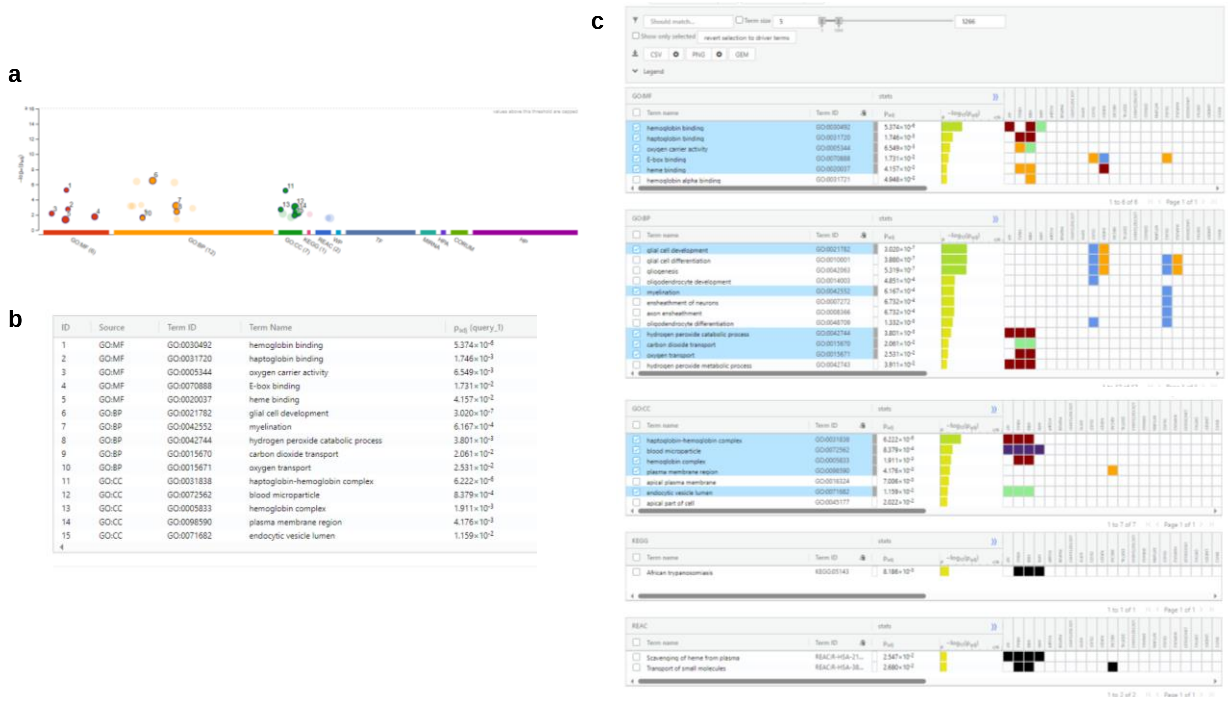

ID	Source	Term ID	Term Name	P_{adj} (query_1)
1	GO:MF	GO:0030492	hemoglobin binding	5.374×10^{-6}
2	GO:MF	GO:0031720	haptoglobin binding	1.746×10^{-3}
3	GO:MF	GO:0005344	oxygen carrier activity	6.549×10^{-3}
4	GO:MF	GO:0070888	E-box binding	1.731×10^{-2}
5	GO:MF	GO:0020037	heme binding	4.157×10^{-2}
6	GO:BP	GO:0021782	glial cell development	3.020×10^{-7}
7	GO:BP	GO:0042552	myelination	6.167×10^{-4}
8	GO:BP	GO:0042744	hydrogen peroxide catabolic process	3.801×10^{-3}
9	GO:BP	GO:0015670	carbon dioxide transport	2.061×10^{-2}
10	GO:BP	GO:0015671	oxygen transport	2.531×10^{-2}
11	GO:CC	GO:0031838	haptoglobin-hemoglobin complex	6.222×10^{-6}
12	GO:CC	GO:0072562	blood microparticle	8.379×10^{-4}
13	GO:CC	GO:0005833	hemoglobin complex	1.911×10^{-3}
14	GO:CC	GO:0098590	plasma membrane region	4.176×10^{-3}
15	GO:CC	GO:0071682	endocytic vesicle lumen	1.159×10^{-2}

Figure S12. Enrichment analysis of CH dataset through G profiler (a) enrichment of select 100 genes from CH dataset (b) 15 capped genes among 100 analyzed: whether the -log10 (p-values) would be capped if >16. (c) Detailed results analyzed by G profiler.

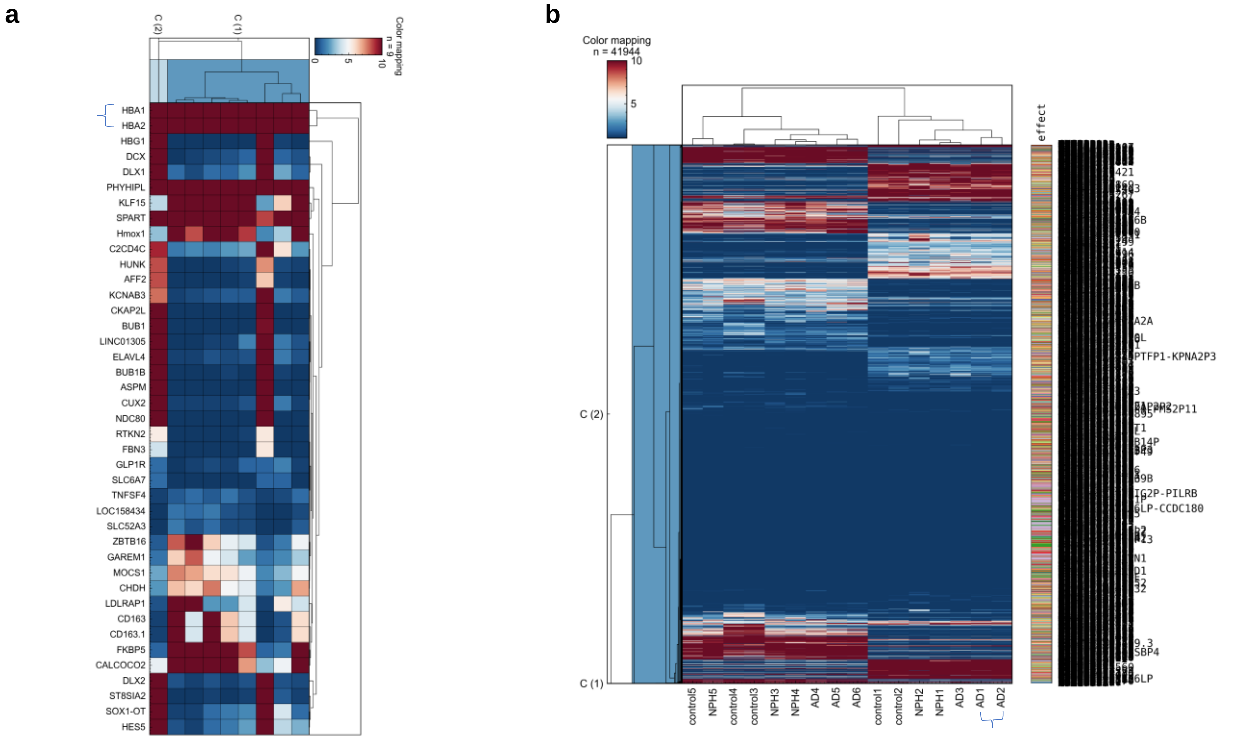

Figure S13. Hierarchical clustering through Instant Clue (a) Gene identity vs. disease group dendrogram summarizing select 40 genes having a different hierarchy as noted by a bracket in blue. Note that HBA1 and HBA2 are in the same cluster while HBG1 is not according to this classification (n=3 per groups of control, CH, and AD). Only select 40 genes sorted by effect size and p value were included to primarily see how genes are clustered. (b) Disease group vs. gene identity dendrogram (transposing columns and rows in a) summarizing all 40,000 genes largely classified into C1 and C2. Note that the disease group is not necessarily classified consistently with diagnosis. For example, AD1 and AD2 are in the same cluster (bracket in blue) while AD3 is not as >40,000 gene readings are included in the analysis (n=5 in control; n=5 in CH; n=6 in AD).

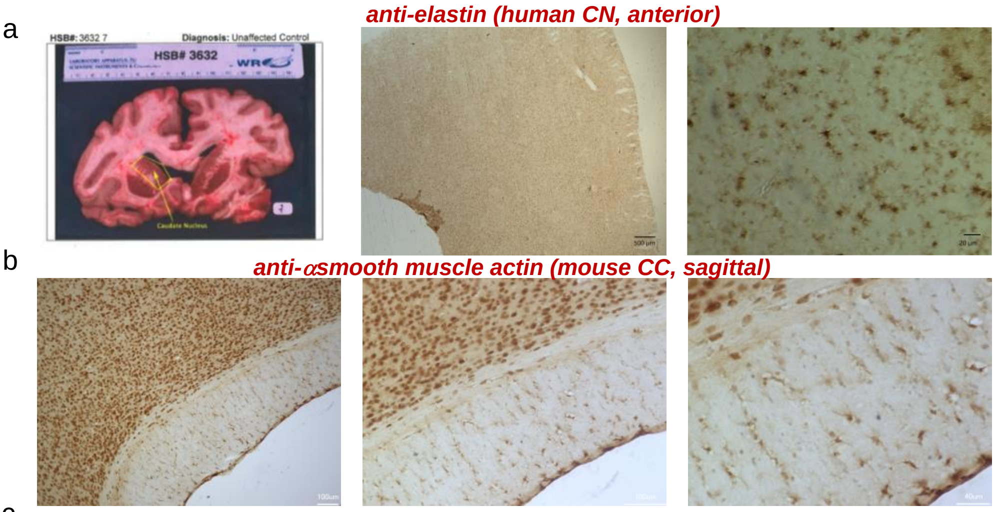

c

ELN by RNA-Seq (human)

CN	Cnt 1	Cnt 2	Cnt 3	Cnt 4	Cnt 5	CH 1	CH 2	CH 3	CH 4	CH 5	AD 1	AD 2	AD 3	AD 4	AD 5	AD 6
FPKM	5.3	19	2.7	2.4	7	5	1.5	4.9	2.9	1.1	6	3.8	5.9	1.4	3.3	4.1

ACTA2 by RNA-Seq (human)

CN	Cnt 1	Cnt 2	Cnt 3	Cnt 4	Cnt 5	CH 1	CH 2	CH 3	CH 4	CH 5	AD 1	AD 2	AD 3	AD 4	AD 5	AD 6
FPKM	0.7	5.8	2.1	3.8	3.2	2.5	2.3	4.9	2.4	2.8	3.4	7.1	3.5	2.9	2.9	3.5

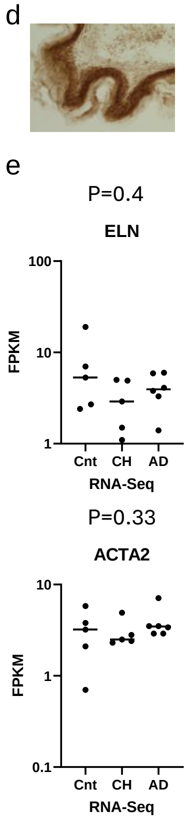

Figure S14. Activities of vascular genes by RNA-Seq and protein localizations via histology (a) Representative micrographs showing localization of elastin in the caudate nucleus (CN) at age 71 years (b) Representative micrographs exhibiting localization of α smooth muscle actin in the CN (arrowhead) and corpus callosum (cc; arrow) in wild type mice at age 20 months (c) Summary of RNA-Seq dataset for ELN (top) and ACTA2 (bottom), which encode elastin and α smooth muscle actin, respectively. Cnt, control; CH, chronic hydrocephalus; AD, Alzheimer's disease (d) localization of elastin (brown/DAB localization) in gut (mouse) (e) Scatter plots shown in tables in c: ELN (top); ACTA2 (bottom). Statistical analysis by Kruskal Wallis test.

Table S1. Two factor characteristics of twenty human genes (Lucas et al., 2021;McKnight et al., 2021;Raines et al., 2022)

Human gene	gene ID	chr*	gene locus	telomere locus (Mb)	gene to telomere (Mb)	Acc. #	A+T (%)	FL*** (bp)
1	LDLRAP1	1	p	25	0	nm_015627	45	3186
2	TNFSF4	1	q	173.35	249	nm_003326	62	3492
3	HPR	16	q	72	90	nm_020995	52	1242
4	NFE2L2	2	q	177.2	241	nm_006164	58	2446
5	Hmox1	22	q	35.3	50	nm_002133	44	1554
6	CD163	12	p	7	0	nm_004244	54	4154
7	HPR	16	q	72	90	nm_020995	52	1242
8	GLP1R	6	p	39	0	nm_002062	52	7018
9	GIPR	19	q	45.6	58.6	nm_000164	45	3683
10	INSR	19	p	7	0	nm_000208	51	9852
11	DPP4	2	q	162	241	nm_001935	59	3573
12	GCGR	17	q	81	83	nm_000160	37	2030
13	PFKM	12	q	48	132	nm_000289	53	3464
14	GAPDH	12	p	6	0	nm_002046	45	1285
15	APOE	19	q	44.9	58.6	nm_000041	32	1166
16	AQP4	18	q	26.8	46	nm_001650	63	3956
17	GLUL	1	q	182.3	248	nm_002065	57	8281
18	HBA1	16	p	0	0	nm_000558	36	577
19	HBA2	16	p	0	0	nm_000517	36	576
20	HBG1	11	p	5	0	nm_000559	50	587

Table S2 Two factor characteristics of incretin genes and other genes compared to Tp53 in 4 species of mice, rats, chimpanzees, and humans.

Human incretin	gene ID	chr*	gene locus (Mb)	telomere locus (Mb)	gene to telomere (Mb)	Acc. #	A+T (%)	FL*** (bp)
1	GLP1R	6	39.1	0	39.1	NM_002062.5	51	6682
2	INSR	19	7.3	0	7.3	NM_000208.4	51	9463
3	DPP4	2	162.1	242.2	80.1	NM_001935.4	59	3573
4	GCGR	17	81.8	83.3	1.5	NM_000160.5	37	2030
5	GIPR	19	45.7	58.6	12.9	NM_000164.4	43	3310
6	LDLRAP1	1	25	0	25	Nm_015627	45	3155
7	NFE2L2	2	177.2	242	65	Nm_006164	58	2446
8	HBA1	16	0.01	0	0.01	Nm_000558	36	577
9	HBG1	11	5	0	5	Nm_000559	50	587
10	HMOX1	22	35	50	15	Nm_002133	44	1554
Ref.	Tp53	17	7.7	0	7.7	Nm_000546	48	2512

Chimp incretin	gene ID	chr*	gene locus (Mb)	telomere locus (Mb)	gene to telomere (Mb)	Acc. #	A+T (%)	FL*** (bp)
1	GLP1R	6	45.0	0	45.0	XM_527380.6	57	16610
2	INSR	19	9.7	0	9.7	XM_016934850.3	51	9468
3	DPP4	2B	64.1	144.4	80.3	XM_515858.6	56	3876
4	GCGR	17	93.4	97.8	4.4	XM_009433521.4	35	5532
5	GIPR	19	48.6	68.1	19.5	XM_054673982.1	42	3604
6	LDLRAP1	1	25	0	25	Xm_016956550	43	3001
7	NFE2L2	2b	79.3	144	64.7	Xm_001145876	56	2699
8	HBA1	16	2.5	0	2.5	Nm_001042626	38	577
9	HBG1	11	9.2	0	9.2	Nm_001135831	53	704
10	HMOX1	22	30.5	52	17	Xm_525579	43	1860
Ref.	Tp53	17	16.4	0	16.4	Xm_001172077	48	2537

Rat incretin	gene ID	chr*	gene locus (Mb)	telomere locus (Mb)	gene to telomere (Mb)	Acc. #	A+T (%)	FL*** (bp)
1	GLP1R	20	9.0	0	9.0	NM_012728.2	46	3065
2	INSR	12	1.3	0	1.3	NM_017071.2	48	5399
3	DPP4	3	47.0	169.0	122.0	NM_012789.2	59	4964
4	GCGR	10	105.8	107.2	1.4	NM_172091.2	46	1893
5	GIPR	1	78.8	260.5	181.7	NM_012714.2	46	2701
6	LDLRAP1	5	146	160	14	Nm_001109271	45	3296
7	NFE2L2	3	60	169	109	Nm_031789	55	2352
8	HBA1	10	15	107	92	Nm_013096	46	557
9	HBG1	1	158	259	101	Nm_172096	55	621
10	HMOX1	19	13	0	13	Nm_012580	49	1600
Ref.	Tp53	10	54	169	115	Nm_030989	49	1792

Mouse incretin	gene ID	chr*	gene locus (Mb)	telomere locus (Mb)	gene to telomere (Mb)	Acc. #	A+T (%)	FL*** (bp)
1	GLP1R	17	31.2	95.3	64.1	NM_021332.2	44	1480
2	INSR	8	3.3	130.1	126.8	NM_010568.3	55	9355
3	DPP4	2	62.2	181.8	119.6	NM_010074.3	59	5268
4	GCGR	11	120.4	122.0	1.6	NM_008101.2	44	1929
5	GIPR	7	18.9	145.0	126.1	NM_001080815.1	44	1792
6	LDLRAP1	4	134	156	22	Nm_145554	44	2671
7	NFE2L2	2	75	180	105	Nm_010902	57	2730
8	HBA1	11	32.2	121	89	Nm_008218	46	569
9	HBG1	NA	NA	NA	NA	NA	NA	NA
10	HMOX1	8	75.8	130	54.2	Nm_010442	49	1634
Ref.	Tp53	11	69.5	122	52.5	Nm_011640	47	1781

Table S3 Inclusion criteria: human postmortem tissues from the NIH NBB

Criteria	Tissue	RNA integrity number (RIN)	Age	Type	Sex	HIV*	HBSAG**	PMInterval***
Include	Cortex, caudate nucleus, cerebellum	7 – 10	≥ 65 yr	Frozen	Male & female	Negative	Negative	< 36 hr
Exclude	elsewhere	<7	< 65 yr	Fixed	-	Positive	Positive	≥ 36 hr

* Human immunodeficiency virus; ** Hepatitis B Surface Antigen Test; *** Postmortem interval

Table S4 Postmortem specimen information

Numbering	Subject ID	Age (years)	Disorder	Sex	Race	Medical History
1	5219	76	Unaffected Control	Female	White	
2	4921	73	Unaffected Control	Female	White	* gall bladder problem
3	4789	72	Unaffected Control	Female	White	
4	5671	78	Unaffected Control	Male	White	
5	s06424	71	Unaffected Control	Female	White	
6	s08544	72	NPH	Female	White	[a]
7	4922	79	NPH	Male	White	[b] TBI
8	3924	79	NPH	Female	White	[c] MS
9	3637	76	NPH	Male	White	[d] cerebral atherosclerosis.
10	21762	77	NPH	Female	African American	[e] vascular dementia
11	1212	72	AD	Female	White	
12	5584	88	AD	Female	White	
13	5501	78	AD	Female	White	
14	6560	67	AD	Female	White	
15	5914	83	AD	Male	White	
16	5683	69	AD	Female	White	

* Donor also with high blood pressure. She collapsed while in the hospital. She was still breathing when found on the floor but with no spontaneous respiration and no cardiac pulse. No further medical history available. "

[a] idiopathic.

[b] personal history of traumatic brain injury (TBI), cerebral atherosclerosis, cerebral infarction (unspecified)

[c] Multiple sclerosis (MS), diagnostic pathology not present

[d] cerebral atherosclerosis, diagnostic pathology not present

[e] vascular dementia without behavioral disturbance

Table S5 Primer sequences for *human* gene transcripts

**Positive control gene** (product size: 249 b); Exon 2<br>(upstream) TCAGCCTTCCCTTACACCAA<br>(downstream) ACAAGTCATCAAAGCCACACA	**GLP1R** (product size: 118 b); Exon 7<br>(upstream) CTCATGCAGTACTGTGTGGC<br>(downstream) TCACGTAGAGCCTGAAGATCC
**LDLRAP1** (product size: 213 b); Exon 2<br>(upstream) TGTTTGCATACATCGCCCAG<br>(downstream) GGTTCCCAGAGATTTCACCC	**GAPDH** (product size: 201 b); Exon 6<br>(upstream) ACCCAGAAGACTGTGGATGG<br>(downstream) TTCTAGACGGCAGGTCAGGT

References for supplementary material

Hastbacka, J., De La Chapelle, A., Kaitila, I., Sistonen, P., Weaver, A., and Lander, E. (1992). Linkage disequilibrium mapping in isolated founder populations: diastrophic dysplasia in Finland. *Nat Genet* 2**,** 204-211.

Lucas, H.B., Mcknight, I., Raines, R., Hijazi, A., Hart, C., Lee, C., Kim, D.G., Li, W., Lee, P.H.U., and Shim, J.W. (2021). Factors Associated with Mutations: Their Matching Rates to Cardiovascular and Neurological Diseases. *Int J Mol Sci* 22.

Mcknight, I., Hart, C., Park, I.H., and Shim, J.W. (2021). Genes causing congenital hydrocephalus: Their chromosomal characteristics of telomere proximity and DNA compositions. *Exp Neurol* 335**,** 113523.

Nusbaum, C., Mikkelsen, T.S., Zody, M.C., Asakawa, S., Taudien, S., Garber, M., Kodira, C.D., Schueler, M.G., Shimizu, A., Whittaker, C.A., Chang, J.L., Cuomo, C.A., Dewar, K., Fitzgerald, M.G., Yang, X., Allen, N.R., Anderson, S., Asakawa, T., Blechschmidt, K., Bloom, T., Borowsky, M.L., Butler, J., Cook, A., Corum, B., Dearellano, K., Decaprio, D., Dooley, K.T., Dorris, L., 3rd, Engels, R., Glockner, G., Hafez, N., Hagopian, D.S., Hall, J.L., Ishikawa, S.K., Jaffe, D.B., Kamat, A., Kudoh, J., Lehmann, R., Lokitsang, T., Macdonald, P., Major, J.E., Matthews, C.D., Mauceli, E., Menzel, U., Mihalev, A.H., Minoshima, S., Murayama, Y., Naylor, J.W., Nicol, R., Nguyen, C., O'leary, S.B., O'neill, K., Parker, S.C., Polley, A., Raymond, C.K., Reichwald, K., Rodriguez, J., Sasaki, T., Schilhabel, M., Siddiqui, R., Smith, C.L., Sneddon, T.P., Talamas, J.A., Tenzin, P., Topham, K., Venkataraman, V., Wen, G., Yamazaki, S., Young, S.K., Zeng, Q., Zimmer, A.R., Rosenthal, A., Birren, B.W., Platzer, M., Shimizu, N., and Lander, E.S. (2006). DNA sequence and analysis of human chromosome 8. *Nature* 439**,** 331-335.

Raines, R., Mcknight, I., White, H., Legg, K., Lee, C., Li, W., Lee, P.H.U., and Shim, J.W. (2022). Drug-Targeted Genomes: Mutability of Ion Channels and GPCRs. *Biomedicines* 10.
